# Simultaneous analysis of several plasticizer classes in different matrices by on-line turbulent flow chromatography-LC–MS/MS

**DOI:** 10.1007/s00216-024-05593-2

**Published:** 2024-10-19

**Authors:** Julio Fernández-Arribas, Sandra Callejas-Martos, Aleix Balasch, Teresa Moreno, Ethel Eljarrat

**Affiliations:** Institute of Environmental Assessment and Water Research (IDAEA)-CSIC, Jordi Girona 18-26, 08034 Barcelona, Spain

**Keywords:** Alternative plasticizers, Face masks, Foodstuffs, Indoor air, Organophosphate esters, Phthalates

## Abstract

The development of methodologies for the determination of plasticizers is essential for assessing the environmental and human impact resulting from the use of plastics. A fast analytical method with on-line purification based on turbulent flow chromatography (TFC) coupled to tandem mass spectrometry (MS–MS) has been developed for the analysis of ten phthalates, four alternative plasticizers (including adipates and citrates), and 20 organophosphate esters (OPEs). The method has been validated for the determination of plasticizers across different matrices. Analytical parameters showed acceptable recoveries ranging between 50 and 125%, RSDs lower than 20%, and mLODs of 0.001–2.08 ng g^−1^ wet weight (ww), 0.002–0.30 ng g^−1^, and 0.001–0.93 ng m^−3^ for foodstuffs, face masks, and ambient air, respectively. These methodologies were applied to foodstuff samples purchased in grocery stores, reusable and self-filtering masks, and indoor air measured in different locations. Plasticizers were detected in all the analyzed samples, with values up to 22.0 μg g^−1^ ww, 6.78 μg g^−1^, and 572 ng m^−3^ for foodstuffs, face masks, and indoor air, respectively. The contribution of each family to the total plasticizer content varied between 1.3 and 87%, 0.5 and 98%, and 0.5 and 65% for phthalates, alternative plasticizers, and OPEs, respectively. These findings highlighted the need for analytical methodologies capable of simultaneously assessing a wide number of plasticizers with minimal extraction steps. This capability is crucial in order to obtain more conclusive insights into the impact of these pollutants on both the environment and human health, arising from different sources of exposure such as foodstuffs, plastic materials, and atmospheric air.

**Graphical Abstract:**

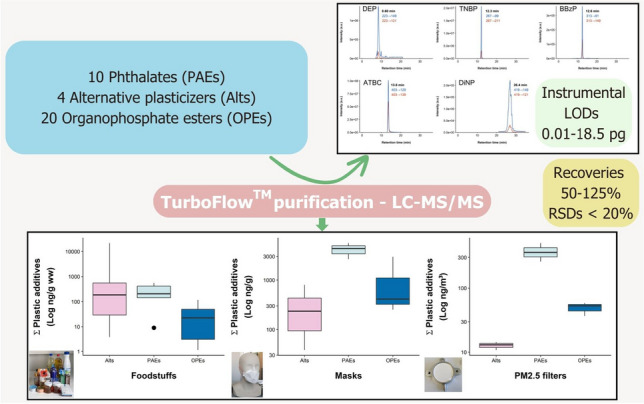

**Supplementary Information:**

The online version contains supplementary material available at 10.1007/s00216-024-05593-2.

## Introduction

Additives such as plasticizers are extensively employed in the production of plastics to improve their physicochemical and mechanical properties. Their significance in the plastic industry has resulted in a usage of 34% of the total additives [1]. Phthalates, also known as ortho-phthalates, such as di(2-ethylhexyl) phthalate (DEHP) or diisononyl phthalate (DiNP) are widely recognized due to their enhancement of flexibility in plastic materials [2]. They are commonly incorporated into plastic polymers, typically constituting a proportion of 10 to 60% of the polymer’s weight [3]. However, the recognition of their toxic effects and subsequent restrictions imposed on diverse products [4] have compelled manufacturing companies to explore alternative compounds that are seemingly less detrimental to human health. Among these alternatives, citric acid esters (CAEs) have emerged as a prominent group of phthalate substitutes [5], alongside 1,2-cyclohexanedicarboxylic acid diisononyl ester (DINCH) or adipates such as di(2-ethylhexyl) adipate (DEHA) [4]. The main CAE, acetyl tributyl citrate (ATBC), is currently the leading substitute due to its non-toxic nature [6], although there is recent evidence suggesting its involvement in the development of fatty liver disease [5]. This exemplification denotes that chemicals perceived as safer may still pose long-term risks to human health and the environment. Given their widespread use, it is crucial to monitor the levels of these compounds over time in different exposure sources, such as foodstuffs, plastic materials involving dermal contact, and the air. This will ease further discussions across various fields, including their impact on waste generation and the performance of risk assessments.

Additionally, organophosphate esters (OPEs) constitute a class of additives that function as dual-purpose components, serving as both flame retardants and plasticizers in a wide range of materials [7]. These compounds are incorporated into plastics, generally within the range of 5 to 20% by weight [1]. Chlorinated OPEs, such as tris(2-chloroisopropyl) phosphate (TCIPP), find predominant use in the manufacturing of polyurethane foams, while other OPEs like tri-n-butyl phosphate (TNBP) or 2-ethylhexyl diphenyl phosphate (EHDPP) are present in lubricants or hydraulic fluids [8]. Nonetheless, OPEs are suspected to be involved in endocrine disruption processes, and mounting evidences suggest that their exposure can lead to modifications in gene expression, a decline in reproductive functions, and other long-term health consequences [9]. Establishing continuous monitoring of these compounds in potential contamination sources will allow for the evaluation of actual exposure and, consequently, the implementation of mitigation measures.

Since these plasticizers are not chemically bonded to plastic materials, their subsequent release into the environment occurs, thereby leading to human exposure through diverse pathways. There is evidence of the presence of phthalates in different food matrices [3], as well as in water, soil, and atmosphere environments [10]. Meanwhile, a limited number of studies have explored the occurrence of alternative plasticizers in certain food items [11, 12], air [13], dust [14, 15], and river waters [16], highlighting the lack of knowledge regarding the actual extent of exposure to these compounds. OPEs have also been detected in various environmental compartments, including air [9], dust [17, 18], fish [19, 20], water [21], packed beverages [22], and foodstuffs [23]. Furthermore, and more recently, the COVID-19 pandemic has highlighted another exposure path via the widespread use of face masks [24–26], the long-term effects of which are currently under scrutiny.

The diverse range of compounds discussed earlier, coupled with the wide variety of matrices and materials in which they are found, poses a challenge in terms of analytical methodologies. The prevailing methods for foodstuff analysis predominantly target either phthalates (and occasionally, alternative plasticizers) or solely OPEs [4, 11, 12, 27–42]. However, there is no study that simultaneously determines both plasticizer groups, mainly due to the available purification methods, which are selective for each group in these kinds of matrices. Concerning face masks, there is a recent study which targeted 6 phthalates and 11 OPEs [43], excluding the assessment of alternatives. In the context of air analysis, one investigation combines 8 phthalates alongside 9 OPEs and 12 polycyclic aromatic hydrocarbons [44], while another study incorporates 7 phthalates and 8 alternatives [13]. Undoubtedly, the limited availability of multi-analyte methods for the detection of these plasticizers across different matrices represents a significant demand that needs to be addressed.

Available studies on phthalates, alternative plasticizers, and OPEs in food have employed ultrasound-assisted extraction (UAE) [40] and conventional solid-phase extraction (SPE) or dispersive SPE as a purification step [31, 33, 38]. Nevertheless, most of the studies have combined a modified QuEChERS method with dispersive SPE [29, 32, 34–37, 39, 41, 42]. Face mask extraction is commonly performed by UAE with a mixture of organic solvents [24, 43, 45], although samples can be dissolved in tetrahydrofuran, followed by a liquid–liquid extraction [46]. In the case of air sampling conducted using quartz fiber filters (QFFs), extraction methods once again involved UAE with organic solvents [9, 13, 47, 48]. It is noteworthy that in these studies, the purification step is not automated, resulting in increased time investment in the sample preparation process. On-line clean-up methods offer an alternative to manual purifications, providing the advantage of automation of this step combined with the chemical analysis [49]. TurboFlow™ technology, based on turbulent flow chromatography (TFC), is one such alternative that has gained prominence in urine samples [50–52] and serum [53], but also in some complex foodstuffs such as royal jelly [49], milk and wine [54], or fish [55]. This application uses large flow rates to combine size exclusion of high molecular weight matrix components with the diffusion of low molecular weight molecules into stationary phase particles. These mechanisms make this technique optimal for the purification of diverse types of matrices, including foodstuffs, filters, and plastic materials. For phthalates and alternatives, the main applications have been focused on the analysis of urine metabolites of phthalates [56–58] and adipates [59, 60]. However, analyses of the parent compounds themselves have not been extensively explored. Specifically, there is no established TurboFlow™ application for analyzing either phthalates or alternatives in foodstuffs, air measurements, and plastic materials. Regarding OPE determination, it has been successfully applied in sediments and fish [19, 61], air and dust samples [17, 62–64], and face masks [24]. These promising results may enable the analysis of other families of plasticizers.

Liquid chromatography (LC) and gas chromatography (GC) coupled to mass spectrometry (MS) are the prevailing instrumental techniques commonly employed for the analysis of plasticizers. GC–MS is the main one for OPE measurements in air [9] and phthalates in foodstuffs [3], although LC–MS/MS has also been used for plasticizers in air [47]. Additionally, some studies in face masks [43] and food samples [12] have been carried out by LC coupled to high-resolution mass spectrometry instruments (HRMS).

The main purpose of this work is to develop a multi-plasticizer analytical method, which includes phthalates, alternative plasticizers, and OPEs in a wide variety of matrices. These matrices include foodstuffs, plastic materials (face masks), and environmental samples (indoor air), recognized as potential sources of environmental and human exposure to plastic additives. Furthermore, in order to achieve a faster, simpler, and automated method, TFC will be adopted as the on-line purification technique, coupled to LC–MS/MS. Analytical parameters such as recoveries, reproducibility, and sensitivity will be estimated to assess the performance of the analytical approach. These parameters will be compared against those obtained from previous publications. Finally, real samples will be analyzed to verify the applicability of the developed methodologies and to assess the extent of plasticizer exposure.

## Materials and methods

### Standards and reagents

Ten phthalates, four alternative phthalates, 20 OPEs, and 18 internal labeled standards (ISs) were employed in this work. Detailed information relative to standards is listed in Table S1. Acetone and hexane were obtained from J.T. Baker (Centre Valley, PA, USA), while methanol, water, ammonium acetate, and formic acid were purchased from Merck (Darmstadt, Germany). Glass wool was provided by Panreac AppliChem (Barcelona, Spain).

### Sample collection and pre-treatment

This study included foodstuffs (low- and high-fat foods), face masks, and indoor air. Food samples were purchased at local grocery stores in Barcelona (Spain). Breakfast cereals, rice, and sweetener packaged in sachets were chosen to represent low-fat food products, while high-fat food items were represented by beef, canned salmon, and natural yogurt. Samples were freeze-dried before analysis. Face masks were obtained from pharmacies and other providers. A cloth reusable mask, and two self-filtering masks of FFP2 and FFP3 types, were selected for analysis. Prior to the extraction of masks, specific components such as ear loops, metal nose strips, valves, or adhesive sticks were removed. Airborne particles of particulate material below 2.5 microns in size (PM2.5) were collected on QFFs (37 mm diameter, PALL) using personal environmental monitors (PEM, SKC) coupled to a Leland Legacy Pump (SKC) at a flow rate of 10 L min^−1^ for 12 h. Indoor environments sampled corresponded to a motorcycle repair garage, an art studio, and a pottery studio.

### Sample extraction

Throughout all the process of sampling and analysis, plastic material was avoided due to potential contamination. All the glass material was previously cleaned without soaps and with ultrapure water, ethanol, and acetone, then heated at 380 °C, wrapped with aluminum foil, and lastly rinsed with an appropriate solvent just before use. However, plasticizer contamination, originating from uncontrollable sources such as ambient air or nitrogen from evaporators, resulted in an inevitable and variable blank signal. Therefore, a laboratory blank was included for every batch of samples.

**Fig. 1:**
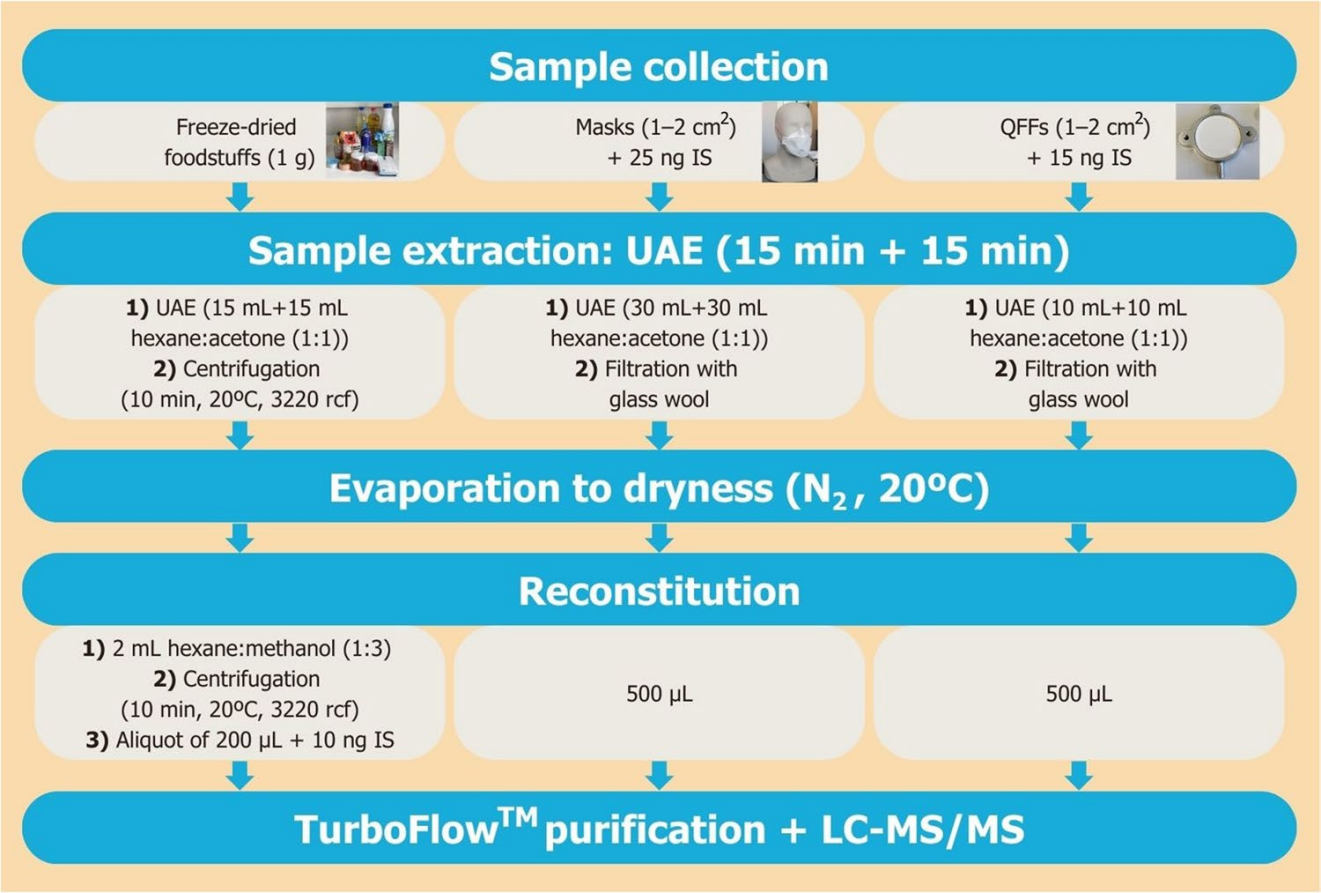
Flowchart of the extraction process for each matrix type

The entire analytical process is summarized in Fig. 1. UAE was the selected extraction technique for all the tested matrices, due to the acceptable outcomes obtained in previous research studies. Foodstuffs extraction was based on Giulivo et al. [65]. One gram of freeze-dried food samples was extracted using 15 mL of hexane:acetone (1:1) mixture. Samples underwent UAE for 15 min, followed by centrifugation at 3220 r.c.f. for 5 min. Both steps were repeated twice, and the resulting extracts were collected and evaporated to dryness at 20 °C under nitrogen stream. A subsequent addition of 2 mL hexane:methanol (1:3) was made, followed by an additional centrifugation step at 3220 r.c.f. for 5 min. An aliquot of 200 µL was then collected and spiked with 10 ng of IS mixture.

Face masks and QFFs were extracted according to OPE procedures previously developed by the authors [24, 64] with minor modifications. Briefly, masks were weighed and cut into pieces measuring approximately 1–2 cm^2^, which were subsequently placed in glass beakers. For QFFs, they were carefully transferred into 50-mL glass centrifuge tubes. Face mask samples were fortified with 25 ng of IS mixture, whereas QFFs were IS-spiked with 15 ng. Afterwards, 30 mL of hexane:acetone (1:1) was added to masks, while 10 mL of the same solvent mixture was added to QFFs. UAE was carried out for 15 min. Extraction process was repeated twice, and both extracts were collected and filtered using glass wool. Solvent was evaporated at 20 °C under a gentle stream of nitrogen. Final extracts were reconstituted in 500 µL of methanol.

### Instrumental analysis

Analytical performance was assessed using a TurboFlow™ TFC-LC–MS/MS provided by Thermo Fisher Scientific (Waltham, MA, USA). This equipment comprised a PAL autosampler, two LC quaternary pumps equipped with a rotor seal to work in Focus mode, a heated-electrospray ionization source (H-ESI), and a TSQ Quantiva triple quadrupole as the measurement instrument. Sample purification was carried out in two combined LC columns, Cyclone™-P (0.5 × 50 mm) and C18-XL (0.5 × 50 mm), while chromatographic separation was performed in a Purosphere Star RP-18 (125 mm × 0.2 mm, particle size 5 µm) analytical column. Mobile phases were performed in gradient elution for both purification (TFC) and analytical (LC) steps, with water (0.1% formic acid) and methanol (0.1% formic acid) solutions for TFC at a flow rate of 0.75 mL min^−1^, and water (0.1% formic acid) and methanol (ammonium acetate 10 mM) solutions for LC at 0.25 mL min^−1^. Gradient composition is summarized in Table S2, with a total run of 36 min. Steps of the analytical process based on Focus mode are included in the Supplementary Information (Fig. S1). Briefly, the sample is loaded onto the combined purification columns, where analytes are retained by diffusion into particle pores. When purification is completed, a backflush is performed in order to transfer analytes to the analytical column. They are focused on the column head using a 200-μL transfer loop containing mobile phase. As analytes are eluted from the analytical column to the MS detector, purification columns are washed to prevent carry-over. Finally, the system is equilibrated, and the transfer loop is refilled for the next injection transfer.

MS/MS measurements were set for spray positive mode at default values that were initially established for OPEs by Giulivo et al. [65]. Those parameters include spray voltage at 3600 V, LC flow rate at 5 μL min^−1^, sheath and auxiliary gas at 5 Arb, sweep gas at 0 Arb, transfer tube temperature at 320 °C, and vaporizer temperature at 50 °C. In this work, individual plasticizer standards encompassing phthalates and alternatives were infused directly into H-ESI so as to optimize declustering potential (DP), collision energy (CE), and two selective reaction monitoring (SRM) transitions for identification and quantification purposes.

### Analytical parameters

An eight-point calibration curve in methanol encompassing all the analytes was prepared, covering a concentration range from 0.1 to 2000 ng mL^−1^ to assess linearity. Additionally, a concentration of 50 ng mL^−1^ was employed for ISs. Standard mixtures were diluted in methanol and stored at − 20 °C. Instrumental limits of detection (iLODs) and quantification (iLOQs) were determined as the minimum analyte quantities that produced signal-to-noise ratios (*S*/*N*) of 3 and 10, respectively. Relative standard deviation (RSD) was the parameter to assess reproducibility, which involved three consecutive injections on the same day (intra-day) and three injections conducted on separate days (inter-day).

For each extraction methodology, recoveries and repeatability were measured at two concentration levels. Foodstuff group was categorized into two groups: low-fat and high-fat samples. Low-fat food samples were fortified with plastic additives at concentrations of 100 ng (low level) and 1000 ng (high level) for each compound. In the case of high-fat food samples, same levels of spiking were applied for phthalates and alternative plasticizers, while for OPEs, concentrations of 10 ng and 100 ng were used for low and high levels, respectively. Regarding face masks, the low-spiked level encompassed 150 ng for phthalates and alternatives, and 25 ng for OPEs, whereas the high-spiked level was set at 1500 ng for phthalates and alternatives, and 250 ng for OPEs. Finally, spiked concentrations for QFFs were 50 ng and 500 ng for phthalates and alternative plasticizers, and 20 ng and 100 ng for OPEs. Estimation of method LODs (mLODs) and LOQs (mLOQs) was conducted using low-level spiked matrices. Four replicates were prepared for each level. Signals from matrix blanks were subtracted from each spiked recovery.

## Results and discussion

### Optimization of sample preparation

As mentioned in the “Introduction” section, UAE with a mixture of organic solvents has been widely employed for extracting plasticizers from face masks and QFFs. While its application in foodstuffs is less frequent, the promising outcomes observed in the specific OPE analysis of fish samples [65], coupled with its agility and reduced extraction of interfering compounds, render it a compromise election. Hence, this previously established OPE method [65] was modified for the inclusion of a larger number of plasticizer classes and diverse matrices. Few modifications have been made to adapt it for phthalates and alternative plasticizers extraction in foodstuffs. Considering the anticipated low OPE concentrations in food samples and the unknown plasticizer levels, a larger dried sample amount of 1 g was selected. However, employing a final volume of 5 mL may lead to analyte dilution, potentially resulting in false negative samples. Due to the increased number of analytes targeted, and therefore improving signal intensity, two different final volumes (1 and 2 mL) were assessed. It is well-known that the presence of matrix effects may lead to ion suppression, particularly in samples with higher fat content, such as meat or fish foodstuffs. With a final reconstitution volume of 1 mL, instrumental noise levels increased, and *S*/*N *ratios for several plasticizers were below 3. Conversely, acceptable *S*/*N* ratios were achieved with 2 mL of final volume. Furthermore, no significant differences in signal were observed between centrifugation times of 10 min and 5 min. Therefore, 5 min was chosen as the optimal time in order to shorten the extraction procedure. With respect to face masks, a reduced volume of hexane:acetone (1:1) amounting to 30 mL was employed, with equivalent results as the original method [24]. Concerning QFFs, the extraction protocol remained unaltered [64].

### Optimization of instrumental conditions

The primary aim of this study is to leverage the extensively validated TurboFlow™ methodology in OPE analysis [65] for other plasticizers. To achieve this goal, a mixture of standards was injected directly into the LC column, both with and without the preceding purification step involving TFC columns. This approach allowed an assessment of analyte separation and the influence of the on-line purification step. Notably, no discernible differences in analytical response were observed between the direct injection into the LC column and the use of the TurboFlow™ purification. Consequently, the on-line purification method proved suitable, as it contributed to obtaining clean extracts free from interfering compounds.

TurboFlow™ offers two operational modes: Quick Elute and Focus modes. Both modes begin with an initial purification step where analytes diffuse into particle pores, while high molecular weight matrix components are separated. In the second step, the flow direction is reversed, transferring the analytes to the elution column for chromatographic resolution. The main difference lies in the pre-concentration at the column head in Focus mode, assisted by a loop filled with mobile phase. This pre-concentration dilutes the sample and retains analytes at the column head, thereby enhancing sensitivity. Consequently, Focus mode was selected for the method optimization. Furthermore, it has been noted that molecules of high weight can be lost during purification step. Thus, the combination of Cyclone™-P and C18-XL purification columns improves recovery rates, in addition to the fact that effectively reduces matrix content due to the diverse polarity range of the stationary phases. Moreover, optimal flow rates in TurboFlow™ columns are critical for efficient matrix removal, but higher flow rates can lead to overpressure drawbacks, analyte loss, and increased solvent consumption. A flow rate of 0.75 mL min^−1^ was determined to be a suitable compromise to establish a turbulent regime. Meanwhile, the transfer time of 2 min (Table S2) was chosen to balance high recovery rates with minimal matrix interferences.

Optimized instrumental parameters and selected transitions are provided in Table 1. Obtained DPs were compatible with the pre-established conditions of spray voltage and mobile phase. After selecting precursor ions for each compound, the three most intense transitions with optimized CEs were identified, and two were chosen based on sensitivity and absence of matrix co-elution. The obtained fragments matched the targeted molecular structures, and no adducts were created. Initially, all transitions were acquired throughout the entire chromatogram with a cycle time of 1 s. However, the inclusion of numerous new transitions of native and IS plasticizers decreased the signal of all compounds, thereby compromising the viability of the method. For enhanced sensitivity, three MS acquisition windows were established between 0.0 and 7.0 min (with 6 compounds, among plasticizers and their ISs), 7.0–21 min (with 36 compounds), and 21–36 (with 11 compounds), employing a cycle time of 0.6 s. Compounds lacking an IS were assigned one within the same window for accurate quantification, considering their similarity in retention time and chemical structure. Linearity was observed within the concentration range of 1 to 2000 ng mL^−1^ for OPEs and most of the newly incorporated compounds, with the exception of di-n-octyl phthalate (DnOP), diisononyl adipate (DINA), and DINCH, which exhibited a range of 10–2000 ng mL^−1^, and DiNP and diisodecyl phthalate (DiDP), with a range of 50–2000 ng mL^−1^. All determination coefficients (*R*^2^) were higher than 0.99. As for repeatability, RSD values were below 20% for both intra-day and inter-day assays. The iLODs for phthalates and alternative plasticizers were in the range from 0.02 to 18.5 injected pg, while the iLOQs ranged from 0.05 to 61.7 injected pg. Available iLODs for these compounds are scarce, and generally higher than values obtained in our study. A GC–MS method applied to PVC medical devices yielded iLODs within the range of 10–60 pg for most of the phthalates, ATBC, and DEHA, and 70–250 pg for DINCH, DiNP, and DiDP [2]. Meanwhile, a LC–MS/MS method targeting phthalates in water showed iLODs ranging from 0.2 to 28.7 pg [66]. Referred to OPEs, the sensitivity (iLODs, 0.01–1.36 pg; iLOQs, 0.03–4.55 pg) remains equal or slightly higher than the previously established by Giulivo et al. [65] in their methodology only focused on OPE analysis. Thus, the expanded range of analytes in the new methodology did not compromise their sensitivity. This outcome has been facilitated through the implementation of MS time windows, improving the sensitivity in T2IPP (0.08 pg of new iLOD versus 1.56 pg), trihexyl phosphate (THP) (0.06 pg versus 0.24 pg), and tris(2-ethylhexyl) phosphate (TEHP) (0.21 pg versus 0.41 pg). Moreover, iLODs were within the same order of magnitude as those observed in OPE methods applied to foodstuffs through LC–MS/MS (iLODs, 0.08–0.48 pg) [34] and GC–MS (iLODs, 0.27–1.04 pg) [38], where a lower number of analytes were targeted. Hence, the characteristics of the methodology made it suitable for application in routine analysis.

**Table 1:**
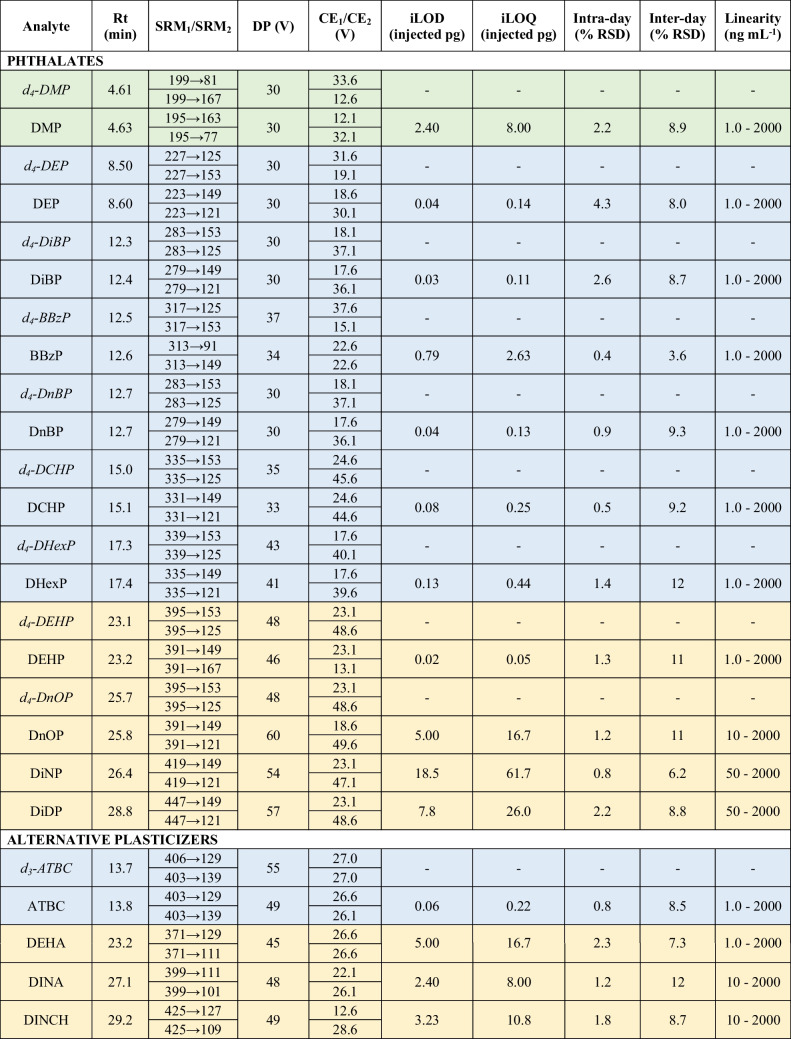

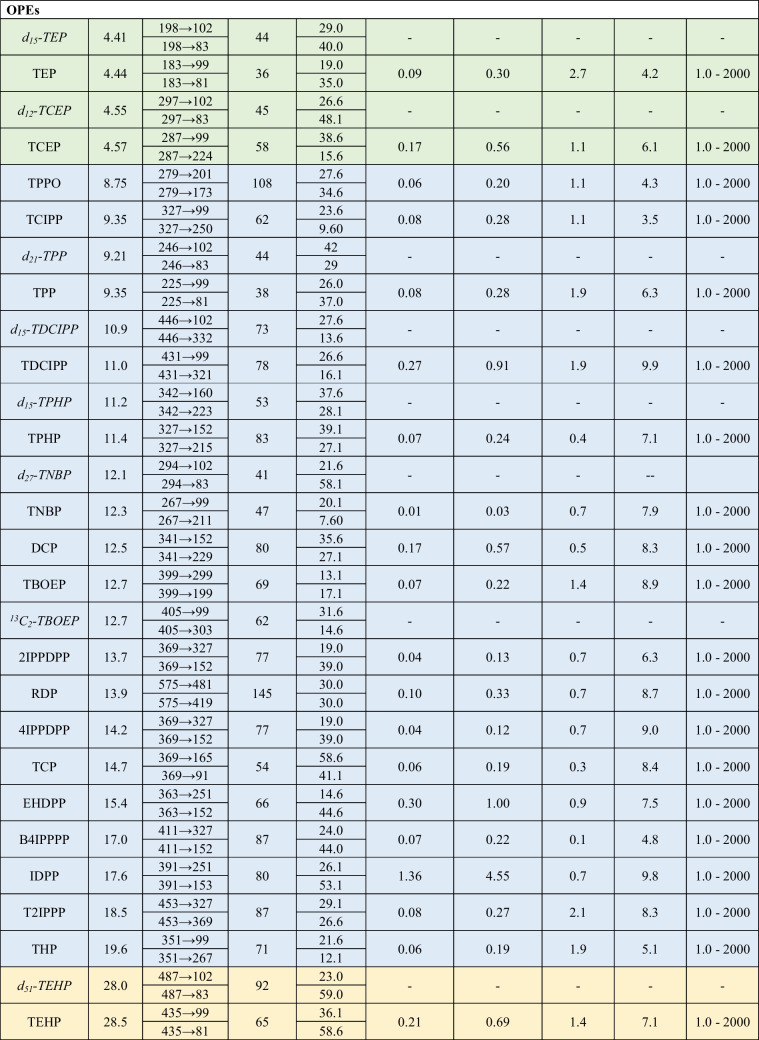
Instrumental TFC-LC–MS/MS parameters, sensitivity, precision, and linearity for each plasticizer analyte (colors correspond to each of the three MS acquisition windows)

Details of compound acronyms can be found in Table S1 of the Supplementary Information

### Analytical parameters

#### Foodstuffs

**Fig. 2:**
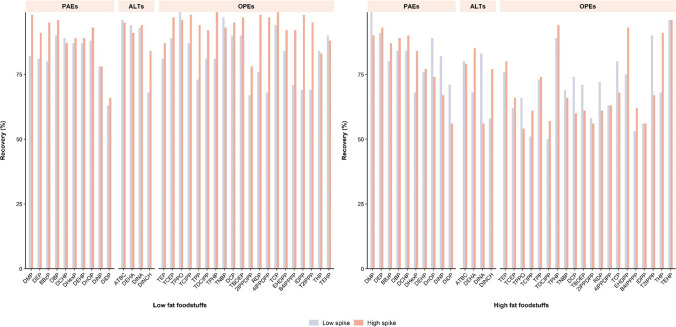
Recoveries of selected plasticizers in foodstuffs analysis (PAEs, phthalates; ALTs, alternative plasticizers; OPEs, organophosphate esters)

A summary of quality parameters related to foodstuffs analysis is presented in Table S3. Due to the influence of the food matrices, chromatographic resolution concerning the two dibutyl phthalate isomers, diisobutyl phthalate (DiBP) and di-n-butyl phthalate (DnBP), was found to be diminished. Consequently, both isomers were quantified as a combined sum. Each category of food was assessed at two concentration levels (Fig. 2). For low-fat food items, recoveries of phthalates and alternative plasticizers exhibited a range of 63–96% for low-spiked level and 66–98% for high-spiked level. Meanwhile, high-fat foodstuff recoveries ranged between 58 and 99% and 56 and 93% for low- and high-spiked levels, respectively. In all instances, RSD values remained consistently below 15%. Regarding sensitivity, mLODs fell within the range of 0.02 to 2.08 ng g^−1 ^wet weight (ww) for low-fat foodstuffs and 0.01 to 1.55 ng g^−1^ ww for high-fat foodstuffs. Limited studies have performed analysis with LC–MS/MS (Table S4). Phthalates in fatty food packaged were analyzed with comparable mLODs ranging from 0.02 to 1.60 ng g^−1^ ww [28]. Another validation of five phthalates, ATBC, and DEHA in cereal-based products provided higher mLODs, which ranged 1.00–50.0 ng g^−1^ ww [11]. More recently, an investigation involving different food composites reported lower mLODs for DEHP, DiNP, and DEHA at 0.10 ng g^−1^ ww each, while diethyl phthalate (DEP) (0.20 ng g^−1^ ww), DnBP (0.10 ng g^−1^ ww), and DINCH (0.30 ng g^−1^ ww) exhibited lower sensitivity than our methodology [40]. In the context of alternative plasticizers, a study of CAEs encompassing various Chinese foodstuffs displayed a higher mLOD for ATBC (0.42 ng g^−1^ ww) compared to the levels obtained in both low-fat (0.03 ng g^−1^ ww) and high-fat foods (0.01 ng g^−1^ ww) [12].

Nonetheless, the prevailing methods for the measurement of phthalates and certain substitutes such as DEHA or DINCH in foodstuffs are based on GC–MS (Table S4). This approach has been coupled with gel permeation chromatography (GPC) as a purification step for the analysis of eight phthalates in food products from Belgium [27]. mLOQs similarities were observed in dimethyl phthalate (DMP) (0.20 compared to 0.28 ng g^−1^ ww of our method), greater sensitivity was noted for DnOP (0.50 versus 0.99 ng g^−1^ ww), and higher values were reported for DEP, butyl benzyl phthalate (BBzP), DiBP, DnBP, dicyclohexyl phthalate (DCHP), and DEHP (1.00–8.00 ng g^−1^ ww) in contrast to our methodology (0.07–3.81 ng g^−1^ ww). In addition, an assay of meat roasted in plastic bags was measured by coupling GC–MS to solid-phase microextraction (SPME), providing mLODs for phthalates and DEHA between 0.01 and 0.18 ng g^−1^ ww [67]. Similarly, a study of dietary exposure to phthalates was performed with gas purge microsyringe extraction (GP-MSE) demonstrating mLODs between 0.14 and 0.38 ng g^−1^ ww [30]. Another diet assessment conducted in Canada brought mLODs for phthalates in the range of 0.99–39.0 ng g^−1^ ww, and a mLOD for DEHA (3.16 ng g^−1^ ww) higher than the mLOQ of our method (2.01 ng g^−1^ ww) [29]. In more recent studies, fish and squid [39] and fast-food items [4] were subjected to extraction for phthalates and DEHA analysis, resulting in method limits ranging 0.50–5.00 ng g^−1^ ww and 1.00–14.0 ng g^−1^ ww, respectively. In the case of fast-food analysis, recoveries were below 30% for DiNP and DINCH, with mLODs (5 ng g^−1^ ww and 10 ng g^−1^ ww, respectively) higher than TurboFlow™ methodology. In recent total diet studies on phthalates conducted in China, two key studies were identified. In one study, 22 phthalates were analyzed, with recoveries ranging from 45 to 136% [68], which is broader than the recovery range obtained in our study (56–99%). In a separate study, six phthalates were examined, with higher LOQs reported (4.3–11.0 ng g^−1^ ww) [69] compared to those achieved in our study (0.04–2.01 ng g^−1^ ww). As regards GC–MS/MS methods, an application to baby foods provided mLOQs ranging 0.03–1.08 ng g^−1^ ww [35], and mLODs of 0.01–5.17 ng g^−1^ ww in edible oils [41]. It is noteworthy that these approaches exhibited sensitivity comparable to our developed method. However, the simplicity, efficiency, and automation offered by the TurboFlow™ technology render it a preferred choice for food analysis.

Regarding OPE quality parameters obtained with our methodology, recovery percentages spanned from 67 to 99% for low-fat foodstuffs and from 50 to 96% for high-fat foodstuffs, all while maintaining RSD values below 13%. Additionally, mLODs ranged between 0.01 and 0.16 ng g^−1^ ww for low-fat foods and between 0.001 and 0.21 ng g^−1^ ww for high-fat food items. Dietary exposure investigations concerning OPEs were carried out predominantly by LC–MS/MS and GC–MS/MS (Table S4). Certain studies only provided information about mLOQs, which fell within the ranges of 0.07–0.42 ng g^−1^ ww [34] and 0.002–62.0 ng g^−1^ ww [32]. These ranges were aligned to the mLOQ values determined in our method (0.004–0.69 ng g^−1^ ww) and in an alternative GC–MS/MS approach (0.07–0.69 ng g^−1^ ww) [38]. Other studies were conducted with methodologies encompassing mLOD ranges of 0.02–0.17 ng g^−1^ ww [33], 0.004–3.30 ng g^−1^ ww [31], and 0.003–0.107 ng g^−1^ ww [36]. Furthermore, a recent advancement by LC-HRMS provided sensitivity values between 0.001 and 0.058 ng g^−1^ ww [42]. Overall, limits derived from those studies resemble our results. Nevertheless, it should be noted that only a limited number of OPEs were targeted in those investigations, whereas our method encompasses an analysis of 34 plastic-related compounds. In addition, reported recovery rates varied across studies. Some, like the methodologies applied in total diet studies from Australia (60–96%) [31], showed ranges similar to those obtained with our method (50–99%). However, other studies have reported different rates, including studies from the USA (32–140%) [33], China (41–136%) [36], (61–136%) [70], and recent studies on take-away foodstuffs (39–121%) [71], all of which exceeded the range observed in our method.

The wide variety of available foodstuffs poses a challenge for the application of the analytical methodology, particularly due to matrix effects. Therefore, an assessment of the IS sensitivity in food matrices was conducted to ensure quality control (Table S5). Recoveries ranged from 60 to 97% across all types of matrices, confirming the robustness of the methodology and highlighting the purification capabilities of TurboFlow™ technology. This approach enabled the simultaneous analysis of diverse compounds, such as citrates, adipates, phthalates, and OPEs, which previously could not be analyzed together in foodstuffs using LC due to their differing chemical nature.

#### Face masks

**Fig. 3:**
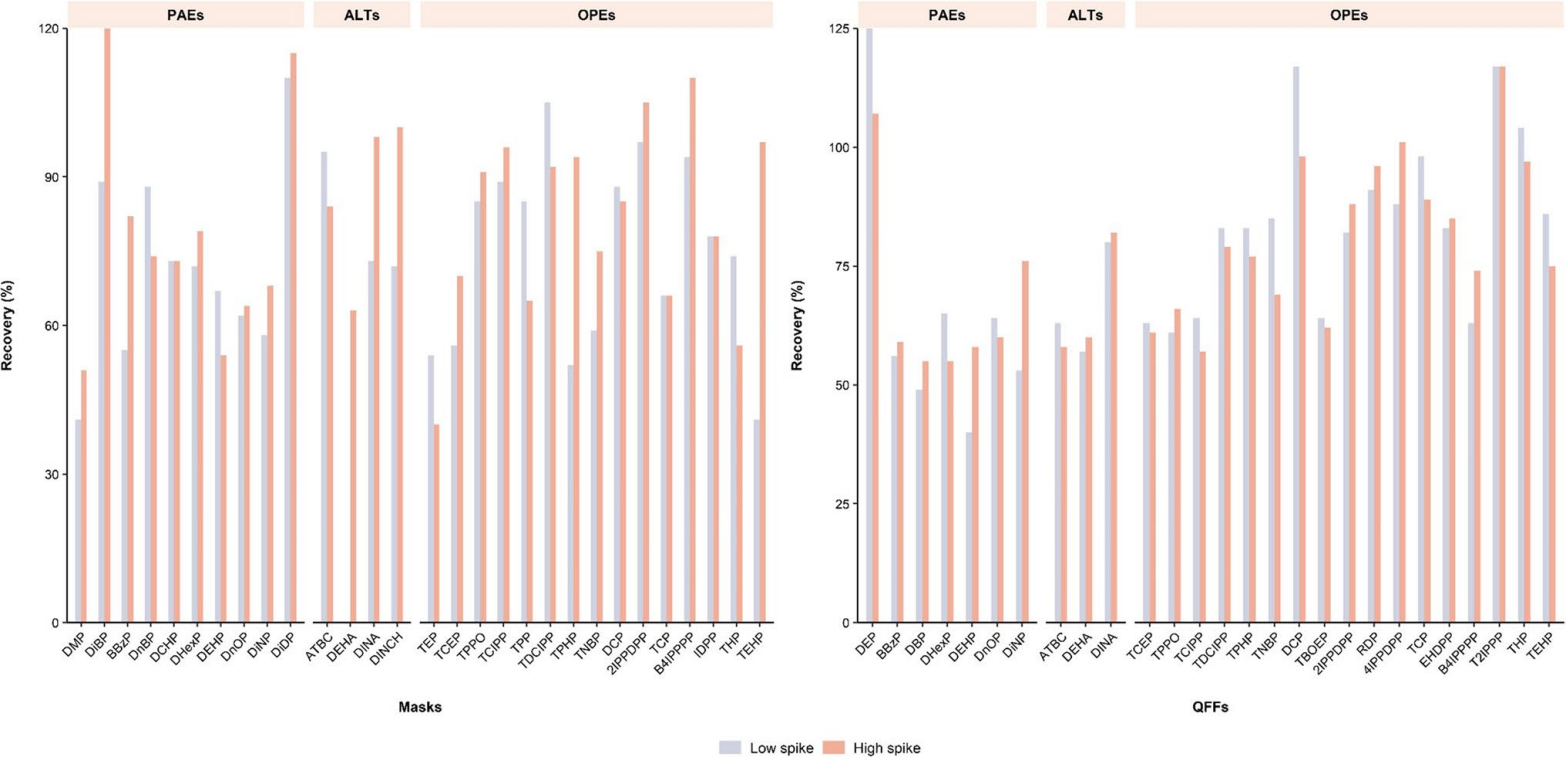
Recoveries of selected plasticizers in face masks and QFFs (PAEs, phthalates; ALTs, alternative plasticizers; OPEs, organophosphate esters)

Face mask quality results are summarized in Table S6. Extraction of plastic additives from face masks resulted in recovery percentages between 52 and 110% for the low-spiked level and between 51 and 120% for the high-spiked level (Fig. 3), with RSD values below 20%, for 29 of the targeted compounds. DMP, triethyl phosphate (TEP), and TEHP exhibited recoveries around 40% at one of the spiked levels. Such recoveries were deemed acceptable, given the inclusion of deuterated ISs in the method for quantification through isotopic dilution, and those compounds had their own deuterated compound. DEP, tris(2-butoxyethyl) phosphate (TBOEP), resorcinol bis(diphenyl phosphate) (RDP), 4-isopropylphenyl diphenyl phosphate (4IPPDPP), and EHDPP were discarded of the methodology due to unacceptable recoveries. mLODs encompass a range of 0.002–0.23 ng g^−1^, while mLOQs ranged from 0.01 to 0.99 ng g^−1^. Given the recent nature of research concerning the environmental and human health implications of the use of face masks, only a limited number of studies have been conducted in this area (Table S4). A single study to date has employed LC-HRMS for the simultaneous analysis of six phthalates and ten OPEs in this context [43], with mLOQs between 0.01 and 0.07 ng g^−1^. Notably, GC–MS was the preferred technique for phthalates analysis, with disparate mLODs ranges: 5.10–26.5 ng g^−1^ [45], 1.10–3.54 ng g^−1^ [72], 10.0–30.0 ng g^−1^ [46], and 0.073–3.41 ng g^−1^ [73]. Additionally, there is a GC-HRMS approach [74] with a wider mLODs range (0.016–10.0 ng sample^−1^) than the one obtained in our developed method (0.003–0.153 ng sample^−1^). To our knowledge, no comprehensive methodologies targeting alternative plasticizers such as ATBC, DEHA, DINA, or DINCH have been reported. Consequently, this method stands as a pioneering approach in the integration of these analytes alongside other plasticizers within the analysis of face masks.

#### Ambient air

QFFs quality results are summarized in Table S6. Similar to the strategy implemented for foodstuffs analysis, DiBP and DnBP isomers were quantified as an aggregated sum in the QFFs methodology. Recovery rates were determined for 27 compounds, spanning from 49 to 125% for the low-spiked level and 55 to 117% for the high-spiked level (Fig. 3), with RSD values remaining below 20%. In this case, 7 compounds (DMP, DCHP, DiDP, DINCH, TEP, tripropyl phosphate (TPP), and isodecyl diphenyl phosphate (IDPP)) were excluded due to low recovery values. DEHP recoveries were considered permissible in this method due to the use of their own deuterated compound, which relies on quantification by isotopic dilution. mLODs for the extraction method ranged from 0.001 to 0.93 ng m^−3^, while mLOQs spanned from 0.004 to 3.10 ng m^−3^. Those levels are compared to recent research that combined the analysis of different additives (Table S4). A GC–MS method developed for outdoor air sampling [75] revealed recovery rates in the range of 45–119% and mLODs between 0.001 and 0.014 ng m^−3^. Moreover, a GC–MS/MS methodology designed for atmospheric particulate matter analysis [76] reported mLODs between 0.003 and 1.13 ng m^−3^ for phthalates, and between 0.003 and 0.16 ng m^−3^ for OPEs. More recent studies based on GC–MS yielded limits in the range of 0.007–0.268 ng m^−3^ for phthalates and 0.003–0.012 ng m^−3^ for OPEs [44], as well as 0.007–0.13 ng m^−3^ for phthalates [48]. Another recent method combined the analysis of phthalates (mLODs, 0.020–13.0 ng m^−3^) with ATBC, DEHA, DINA, and DINCH (mLODs, 0.014–4.6 ng m^−3^) [13]. Additionally, an LC–MS/MS-based approach targeting phthalates and DEHA [47] reported mLODs from 0.03 to 1.15 ng m^−3^. In summary, available methods consistently provided comparable outcomes to our developed methodology, which encompassed a larger number of analytes than most studies. Furthermore, it provides a new methodology for monitoring alternative additives, which study will be under scrutiny.

### Application to real samples

**Fig. 4:**
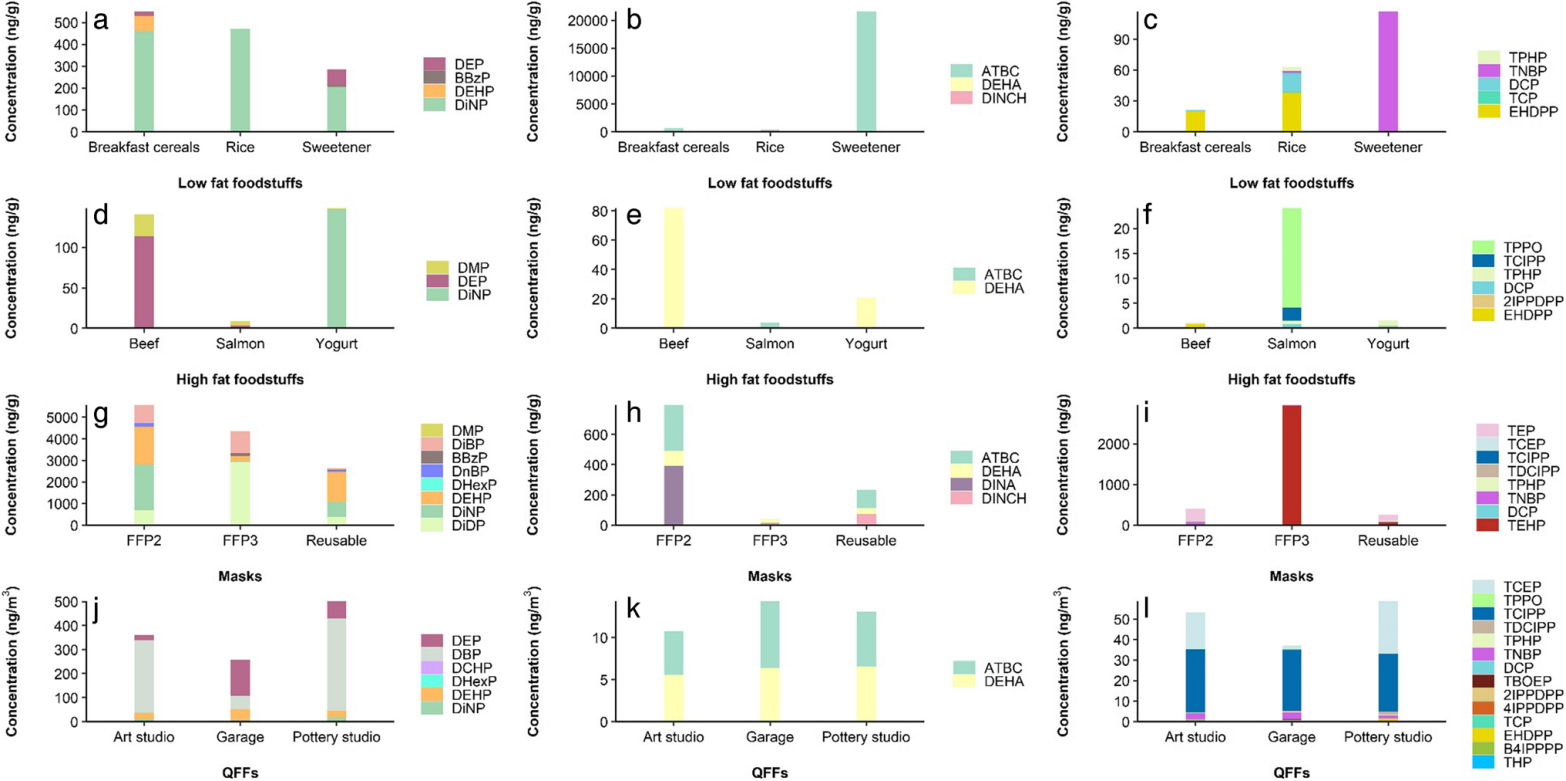
Plasticizer levels found in low-fat foodstuffs (**a**, **b**, **c**), high-fat foodstuffs (**d**, **e**, **f**), face masks (**g**, **h**, **i**), and QFFs (**j**, **k**, **l**) (DBP = sum of both isomers, DiBP and DnBP levels)

**Fig. 5:**
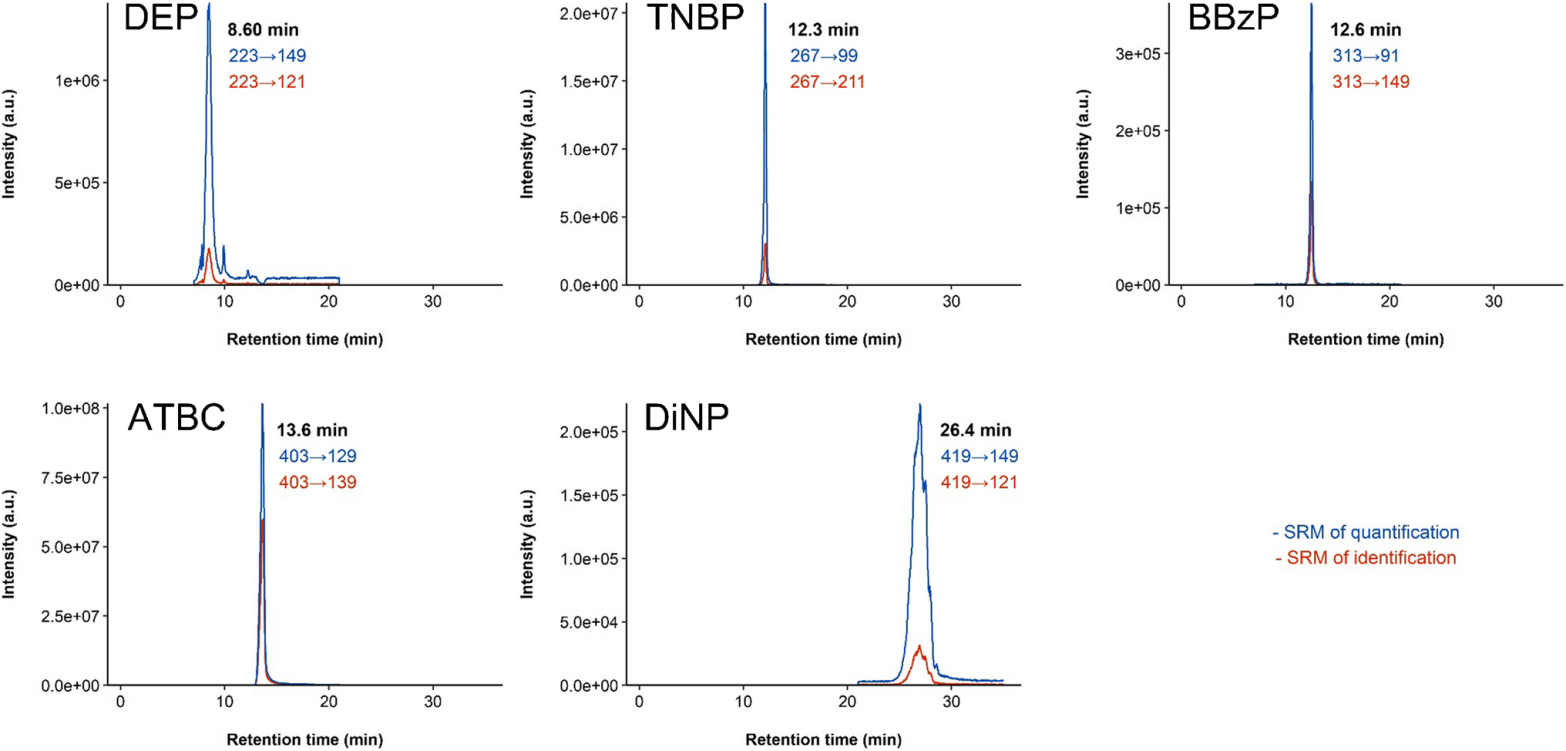
Chromatograms of 5 plasticizers found in a sweetener sample

Samples corresponding to the different matrices for which the methods have been developed were subjected to analysis of plastic additives. Figure 4 presents the plasticizer levels in three low-fat foodstuffs, three high-fat foodstuffs, three face masks, and three indoor air environments (individual results are provided in Table S7). Exemplified in Fig. 5 is the chromatographic profile of the sweetener sample, where 5 plasticizers were detected and quantified.

Concerning foodstuff samples, 16 out of the 34 targeted plasticizers were identified. Notably, results revealed that low-fat food items exhibit major additive levels compared to those with higher fat content. In specific terms, phthalate concentrations spanned from 1.3 to 49% of total additives in low-fat samples, a range that expanded to 25–87% in high-fat items. In the case of alternative plasticizers and OPEs, the observed ranges were 44–98% and 0.5–6.5%, respectively, for low-fat foodstuffs. These percentages changed to 10–37% for alternatives and 0.5–65% for OPEs in high-fat samples. DiNP predominantly appears in breakfast cereals, rice, and sweetener (Fig. 4a), with concentrations ranging from 205 to 459 ng g^−1^ ww. Particularly significant is the presence of ATBC (21.6 μg g^−1^ ww, Fig. 4b) in the sweetener sample (Fig. 5), highlighting the increasing use of alternative plasticizers. Moreover, the sweetener sample stands out due to its dominant TNBP content compared to breakfast cereals and rice (Fig. 4c), where EHDPP was detected at 19.9 and 38.1 ng g^−1^ ww, respectively. With regard to high-fat foods, beef showed noteworthy levels of DEP (114 ng g^−1^ ww), and yogurt presented high DiNP levels (147 ng g^−1^ ww, Fig. 4d), while DEHA is also detected in both samples (81.9 and 20.7 ng g^−1^ ww, respectively, Fig. 4e). The salmon sample was particularly high in OPE levels (Fig. 4f), with the main contributor being triphenylphosphine oxide (TPPO) (20.0 ng g^−1^ ww) and TCIPP (2.59 ng g^−1^ ww).

As regards face masks, 20 out of 29 compounds were detected. The relative contribution of phthalates to total additives was in the range between 59 and 84%. In contrast, alternative additives and OPEs exhibited 0.5–12% and 6.1–40%, respectively. The FFP2 sample exhibited the highest levels of phthalates (Fig. 4g) and alternative plasticizers (Fig. 4h), with remarkable concentrations of DiNP (2094 ng g^−1^), DEHP (1766 ng g^−1^), DINA (391 ng g^−1^), and ATBC (304 ng g^−1^). In contrast, the FFP3 sample notably presented heightened levels of DiDP (2933 ng g^−1^) and TEHP (2938 ng g^−1^, Fig. 4i), while reusable masks demonstrated the lowest levels of plasticizers across each group.

The analysis of indoor air samples provided insight into the exposure to plasticizers over different commercial locations, with the detection of 22 out of 27 targeted additives, and contribution ranges of 83–87%, 2.3–4.6%, and 10–13% for phthalates, alternatives, and OPEs, respectively. DEP and DBP (sum of isomers) emerged as the most prevalent phthalate contributors in the selected spots (Fig. 4j), with levels ranging from 22.1 to 385 ng m^−3^. The disparity is evident when considering alternative plasticizers (Fig. 4k), where the levels reached a maximum of 14.3 ng m^−3^. This observation underscores the prevailing presence of conventional plasticizers in these work environments. Notably, OPEs (Fig. 4l) exhibited the widest variety of identified compounds, with levels ranging from 36.9 to 58.6 ng m^−3^. Among these, tris(2-chloroethyl) phosphate (TCEP), TCIPP, and TNBP emerged as remarkable contributors.

The obtained results demonstrated the presence of alternative plasticizers across different exposure sources, including ingestion (foodstuffs), dermal contact (face masks), and inhalation (ambient air). In fact, the presence of alternative plasticizers such as ATBC in a sweetener sample emphasizes the ubiquity of this compound in the environment. Moreover, the detection of traditional phthalates in indoor air and certain OPEs in face masks is a growing concern due to their potential long-term health effects. The American Environmental Protection Agency (USEPA) [77] and the European Union [78] have established criteria for the maximum tolerable levels of some compounds. Although individual samples did not exceed these thresholds, the cumulative exposure might reach those levels.

## Conclusions

A new methodology with on-line purification based on TFC coupled to LC–MS/MS has been developed for the analysis of up to 34 plastic additives in a wide variety of matrices with very different characteristics, including foodstuffs, plastic materials (face masks), and indoor air. Analytical parameters such as recoveries, reproducibility, and sensitivity were estimated to assess the method quality. As a result, acceptable recoveries ranging from 50 to 99% for foodstuffs, from 50 to 120% for face masks, and from 50 to 125% for QFF were exhibited, always with RSDs below 20%. With regard to sensitivity, mLODs ranged from 0.001 to 2.08 ng g^−1^ ww, from 0.002 to 0.30 ng g^−1^, and from 0.001 to 0.93 ng m^−3^ for foodstuffs, face masks, and ambient air, respectively. These values were comparable or lower to previous published studies, with the notable advantage of this methodology being novel in the simultaneous determination of diverse plasticizer groups with an on-line purification step. Finally, developed methodologies were applied to different foodstuff samples, cloth reusable masks, FFP2 and FFP3 masks, and indoor air from a motorcycle garage, and art and pottery studios. The three plasticizer families were detected in all analyzed samples, with phthalates and alternatives exhibiting the highest levels. The notable presence of alternative additives, for which there are limited analytical methods and data on their occurrence, underscores the importance of the developed method for future monitoring studies.

## Supplementary Information

Below is the link to the electronic supplementary material.

Supplementary file1 (DOCX 318 KB)
