# Supplementary material for: Simultaneous analysis of several plasticizer classes in different matrices by on-line turbulent flow chromatography-LC–MS/MS
Source: Anal Bioanal Chem. 2024 Oct 19;416(29):6957–72. doi: 10.1007/s00216-024-05593-2 (PMC11579108; doi:10.1007/s00216-024-05593-2)
Supplement: Supplementary file 1 — Supplementary file1 (DOCX 318 KB) [file 216_2024_5593_MOESM1_ESM.docx]

***Supplementary information***

**Simultaneous analysis of several plasticizer classes in different matrices by on-line turbulent flow chromatography-LC-MS/MS**

**Analytical and Bioanalytical Chemistry**

Julio Fernández-Arribas^*^, Sandra Callejas-Martos, Aleix Balasch, Teresa Moreno, Ethel Eljarrat^*^

Institute of Environmental Assessment and Water Research (IDAEA)-CSIC, Jordi Girona 18-26, 08034 Barcelona (Spain)

*Corresponding authors. E-mail addresses: jfaqam@cid.csic.es (J. Fernández-Arribas), ethel.eljarrat@idaea.csic.es (E. Eljarrat).

**Supply materials**

**Table S1.** Native and internal labelled standards (ISs) of plastic additives used in this study.

**Table S2.** TFC-LC methodology for purification (pump 1) and chromatographic separation (pump 2) of plastic additives.

**Table S3.** Recoveries, relative standard deviations (RSDs), and sensitivity parameters of selected plasticizers in foodstuffs analysis.

**Table S4.** Cleanup methodologies, instrumental techniques, and method limits of detection (mLODs) and quantification (mLOQs) of different groups of plasticizers in foodstuffs, face masks, and ambient air from other published studies.

**Table S5.** Internal standard (IS) recovery ranges (%) in the different matrices analyzed.

**Table S6.** Recoveries, relative standard deviations (RSDs), and sensitivity parameters of selected plasticizers in face masks and QFFs.

**Table S7.** Plasticizer results from the developed method applied to selected real samples.

**Figure S1.** TurboFlow™ workflow description of the Focus mode.

**Table S1.** Native and internal labelled standards (ISs) of plastic additives used in this study.

| **Acronym** | **Name** | **CAS number** | **Molecular weight (g/mol)** | **Manufacturer** | **IS** |
| --- | --- | --- | --- | --- | --- |
| **PHTHALATES** | | | | | |
| DMP | Dimethyl phthalate | 131-11-3 | 194.2 | AS | d_4_-DMP |
| DEP | Diethyl phthalate | 84-66-2 | 222.2 | AS | d_4_-DEP |
| DiBP | Diisobutyl phthalate | 84-69-5 | 278.4 | AS | d_4_-DiBP |
| BBzP | Butyl benzyl phthalate | 85-68-7 | 312.4 | AS | d_4_-BBzP |
| DnBP | Di-n-butyl phthalate | 84-74-2 | 278.4 | AS | d_4_-DnBP |
| DCHP | Dicyclohexyl phthalate | 84-61-7 | 330.4 | AS | d_4_-DCHP |
| DHexP | Dihexyl phthalate | 84-75-3 | 334.5 | AS | d_4_-DHexP |
| DEHP | Di(2-ethylhexyl) phthalate | 117-81-7 | 390.6 | AS | d_4_-DEHP |
| DnOP | Di-n-octyl phthalate | 117-84-0 | 390.6 | AS | d_4_-DnOP |
| DiNP | Diisononyl phthalate | 28553-12-0 | 418.6 | AS | d_4_-DnOP |
| DiDP | Diisodecyl phthalate | 26761-40-0 | 446.7 | AS | d_4_-DnOP |
| **ALTERNATIVE PLASTICIZERS** | | | | | |
| ATBC | Acetyl tributyl citrate | 77-90-7 | 402.5 | AS | d_3_-ATBC |
| DEHA | Di(2-ethylhexyl) adipate | 103-23-1 | 370.6 | AS | d_4_-DEHP |
| DINA | Diisononyl adipate | 33703-08-1 | 398.3 | DE | d_4_-DnOP |
| DINCH | 1,2-cyclohexanedicarboxylic acid, 1,2-diisononyl ester | 166412-78-8 | 424.7 | DE | d_4_-DEHP |
| **OPEs** | | | | | |
| TEP | Triethyl phosphate | 78-40-0 | 182.1 | M | d_15_-TEP |
| TCEP | Tris(2-chloroethyl) phosphate | 115-96-8 | 285.5 | SC | d_12_-TCEP |
| TPPO | Triphenylphosphine oxide | 791-28-6 | 278.3 | M | d_15_-TDCIPP |
| TCIPP | Tris(2-chloroisopropyl) phosphate | 13674-84-5 | 327.6 | SC | d_15_-TDCIPP |
| TPP | Tripropyl phosphate | 513-08-6 | 224.2 | M | d_21_-TPP |
| TDCIPP | Tris(1,3-dichloro-2-propyl) phosphate | 13674-87-8 | 430.9 | M | d_15_-TDCIPP |
| TPHP | Triphenyl phosphate | 115-86-6 | 326.3 | M | d_15_-TPHP |
| TNBP | Tri-n-butyl phosphate | 126-73-8 | 266.3 | M | d_27_-TNBP |
| DCP | Diphenyl cresyl phosphate | 26444-49-5 | 340.3 | M | d_27_-TNBP |
| TBOEP | Tris(2-butoxyethyl) phosphate | 78-51-3 | 398.5 | SC | ^13^C_6_-TBOEP |
| 2IPPDPP | 2-isopropylphenyl diphenyl phosphate | 64532-94-1 | 368.4 | WL | ^13^C_6_-TBOEP/ d_15_-TPHP^*^ |
| RDP | Resorcinol bis(diphenyl phosphate) | 57583-54-7 | 574.5 | CIL | ^13^C_6_-TBOEP/ d_15_-TPHP^*^ |
| 4IPPDPP | 4-isopropylphenyl diphenyl phosphate | 55864-04-5 | 368.4 | WL | ^13^C_6_-TBOEP/ d_15_-TPHP^*^ |
| TCP | Tricresyl phosphate | 1330-78-5 | 368.4 | DE | ^13^C_6_-TBOEP/ d_15_-TPHP^*^ |
| EHDPP | 2-ethylhexyl diphenyl phosphate | 1241-94-7 | 362.4 | AS | ^13^C_6_-TBOEP/ d_15_-TPHP^*^ |
| B4IPPPP | Bis(4-isopropylphenyl)phenyl phosphate | 55864-07-8 | 410.4 | WL | ^13^C_6_-TBOEP/ d_15_-TPHP^*^ |
| IDPP | Isodecyl diphenyl phosphate | 29761-21-5 | 390.5 | AS | ^13^C_6_-TBOEP/ d_15_-TPHP^*^ |
| T2IPPP | Tris(2-isopropylphenyl) phosphate | 64532-95-2 | 452.5 | CH | ^13^C_6_-TBOEP/ d_15_-TPHP^*^ |
| THP | Trihexyl phosphate | 2528-39-4 | 350.5 | SC | ^13^C_6_-TBOEP/ d_15_-TPHP^*^ |
| TEHP | Tris(2-ethylhexyl) phosphate | 78-42-2 | 434.6 | SC | d_51_-TEHP |
| **IS** | | | | | |
| d_4_-DMP | Dimethyl phthalate-d_4_ | 93951-89-4 | 198.2 | AS | - |
| d_4_-DEP | Diethyl phthalate-d_4_ | 93952-12-6 | 226.2 | AS | - |
| d_4_-DiBP | Diisobutyl phthalate-d_4_ | 358730-88-8 | 282.4 | AS | - |
| d_4_-BBzP | Butyl benzyl phthalate-d_4_ | 93951-88-3 | 316.3 | A2S | - |
| d_4_-DnBP | Di-n-butyl phthalate-d_4_ | 93952-11-5 | 282.4 | AS | - |
| d_4_-DCHP | Dicyclohexyl phthalate-d_4_ | 358731-25-6 | 334.4 | AS | - |
| d_4_-DHexP | Dihexyl phthalate-d_4_ | 1015854-55-3 | 338.5 | AS | - |
| d_4_-DEHP | Di(2-ethylhexyl) phthalate-d_4_ | 93951-87-2 | 394.6 | AS | - |
| d_4_-DnOP | Di-n-octyl phthalate-d_4_ | 93952-13-7 | 394.6 | AS | - |
| d_3_-ATBC | Acetyl tributyl citrate-d_3_ | 1794753-49-3 | 405.5 | CDN | - |
| d_15_-TEP | Triethyl phosphate-d_15_ | 135942-11-9 | 197.3 | CIL | - |
| d_12_-TCEP | Tris(2-chloroethyl) phosphate-d_12_ | 1276500-47-0 | 297.6 | CIL | - |
| d_21_-TPP | Tripropyl phosphate-d_21_ | 1219794-92-9 | 245.4 | CIL | - |
| d_15_-TDCIPP | Tris(1,3-dichloro-2-propyl) phosphate-d_15_ | 1447569-77-8 | 446.0 | CIL | - |
| d_15_-TPHP | Triphenyl phosphate-d_15_ | 1173020-30-8 | 341.4 | CIL | - |
| d_27_-TNBP | Tri-n-butyl phosphate-d_27_ | 61196-26-7 | 293.5 | CIL | - |
| ^13^C_6_-TBOEP | Tris(2-butoxy-[^13^C_6_]-ethyl) phosphate | - | 404.0 | WL | - |
| d_51_-TEHP | Tris(2-ethylhexyl) phosphate-d_51_ | 1259188-37-8 | 486.0 | TRC | - |

SC: Santa Cruz Biotechnology (Santa Cruz, CA, USA); M: Merck (Darmstadt, Germany); WL: Wellington Laboratories Inc. (Guelph, ON, Canada); CIL: Cambridge Isotope Laboratories Inc. (Andover, MA, USA); DE: Dr. Ehrenstorfer (Augsburg, Germany); AS: AccuStandard (New Haven, CT, USA); CH: Chiron (Trondheim, Norway); TRC: Toronto Research Chemicals (Toronto, ON, Canada); A2S: Analytical Standard Solutions (Saint-Jean-d’Illac, France); CDN: CDN Isotopes (Pointe-Claire, QC, Canada).

*IS used in the analysis of face masks and ambient air.

**Table S2.** TFC-LC methodology for purification (pump 1) and chromatographic separation (pump 2) of plastic additives.

|  | | | **Pump 1: Load Pump (Turboflow)** | | | | | **Pump 2: Elute Pump (Chromatographic)** | | | | |
| --- | --- | --- | --- | --- | --- | --- | --- | --- | --- | --- | --- | --- |
|  |  |  | Injection volume: 20μL | | | | |  | | | | |
|  |  |  | Purification columns: CycloneTM-P (0.5x50mm)  C18-XL (0.5x50mm) | | | | | Analytical column: Purosphere STAR RP-18 (125mmx2.0mm) | | | | |
|  |  |  | Solvent A: Water + 0.1% formic acid  Solvent B: Methanol+ 0.1% formic acid | | | | | Solvent A: Water + 0.1% formic acid  Solvent B: Methanol+ ammonium acetate 10 mM | | | | |
| Step | Loop | Start  (min) | Flow  (ml/min) | Gradient | A% | B% | Description | Flow | Gradient | A% | B% | Description |
| 1 | out | 0.00 | 0.75 | Step | 98 | 2 | Sample loading into the TF column | 0.25 | Step | 50 | 50 | Analytical column conditioning |
| 2 | in | 2.00 | 0.13 | Step | - | 100 | Analyte transfer to analytical column | 0.12 | Step | 100 | - | Analyte transfer to analytical column |
| 3 | in | 4.00 | 0.75 | Step | - | 100 | TurboFlow^TM^ column cleaning | 0.25 | Step | 50 | 50 | LC separation |
| 4 | in | 5.00 | 0.75 | Step | - | 100 | TurboFlow^TM^ column and loop cleaning | 0.25 | Ramp | 20 | 80 | LC separation |
| 5 | in | 7.00 | 0.75 | Step | - | 100 | TurboFlow^TM^ column and loop cleaning | 0.25 | Step | 20 | 80 | LC separation |
| 6 | in | 8.00 | 0.75 | Step | - | 100 | TurboFlow^TM^ column and loop cleaning | 0.25 | Ramp | 10 | 90 | LC separation |
| 7 | out | 13.00 | 0.75 | Step | - | 100 | TurboFlow^TM^ column conditioning | 0.25 | Step | 10 | 90 | Analytical column cleaning |
| 8 | out | 21.00 | 0.40 | Step | - | 100 | TurboFlow^TM^ column conditioning | 0.25 | Ramp | - | 100 | Analytical column cleaning |
| 9 | out | 26.00 | 0.40 | Step | - | 100 | TurboFlow^TM^ column conditioning | 0.25 | Step | - | 100 | Analytical column cleaning |
| 10 | out | 35.00 | 0.40 | Step | - | 100 | TurboFlow^TM^ column conditioning | 0.25 | Ramp | 50 | 50 | Analytical column conditioning |
| 11 | out | 36.00 | 0.75 | Step | 98 | 2 | TurboFlow^TM^ column conditioning | 0.25 | Step | 50 | 50 | Analytical column conditioning |

**Table S3.** Recoveries, relative standard deviations (RSDs), and sensitivity parameters of selected plasticizers in foodstuffs analysis.

| **Analyte** | **Low fat foodstuffs** | | | | | | **High fat foodstuffs** | | | | | |
| --- | --- | --- | --- | --- | --- | --- | --- | --- | --- | --- | --- | --- |
|  | **Low spike - 100 ng**  **(n = 4)** | | **High spike - 1000 ng (n = 4)** | | **mLOD (ng g^-1^ ww)** | **mLOQ (ng g^-1^ ww)** | **Low spike***  **(n = 4)** | | **High spike***  **(n = 4)** | | **mLOD (ng g^-1^ ww)** | **mLOQ (ng g^-1^ ww)** |
|  | **Recovery (%)** | **RSD (%)** | **Recovery (%)** | **RSD (%)** |  |  | **Recovery (%)** | **RSD (%)** | **Recovery (%)** | **RSD (%)** |  |  |
| **PHTHALATES** | | | | | | | | | | | | |
| DMP | 82 | 3.1 | 98 | 6.0 | 0.08 | 0.28 | 99 | 7.8 | 90 | 8.3 | 0.11 | 0.38 |
| DEP | 81 | 15 | 91 | 4.8 | 0.04 | 0.15 | 91 | 7.2 | 93 | 7.3 | 0.05 | 0.17 |
| BBzP | 80 | 8.9 | 95 | 9.9 | 0.06 | 0.18 | 80 | 11 | 87 | 7.4 | 0.07 | 0.24 |
| DiBP+DnBP | 90 | 10 | 96 | 11 | 0.03 | 0.09 | 84 | 9.4 | 89 | 10 | 0.04 | 0.15 |
| DCHP | 89 | 3.3 | 87 | 9.5 | 0.02 | 0.07 | 84 | 1.9 | 90 | 7.2 | 0.03 | 0.09 |
| DHexP | 87 | 4.0 | 89 | 8.9 | 0.05 | 0.17 | 68 | 5.5 | 84 | 8.1 | 0.13 | 0.43 |
| DEHP | 87 | 7.2 | 89 | 6.9 | 1.14 | 3.81 | 76 | 11 | 77 | 8.7 | 0.10 | 0.34 |
| DnOP | 88 | 9.2 | 93 | 2.9 | 0.30 | 0.99 | 89 | 6.9 | 74 | 6.0 | 0.06 | 0.21 |
| DiNP | 78 | 5.6 | 78 | 5.0 | 2.08 | 6.92 | 82 | 12 | 67 | 9.1 | 0.50 | 1.66 |
| DiDP | 63 | 8.8 | 66 | 6.7 | 0.53 | 1.76 | 71 | 9.9 | 56 | 7.7 | 1.55 | 5.16 |
| **ALTERNATIVE PLASTICIZERS** | | | | | | | | | | | | |
| ATBC | 96 | 5.1 | 95 | 9.3 | 0.03 | 0.09 | 80 | 6.1 | 79 | 6.2 | 0.01 | 0.04 |
| DEHA | 94 | 6.1 | 91 | 4.0 | 0.60 | 2.01 | 68 | 11 | 85 | 6.7 | 0.01 | 0.05 |
| DINA | 93 | 7.5 | 94 | 5.6 | 0.22 | 0.73 | 83 | 9.0 | 56 | 8.1 | 0.09 | 0.31 |
| DINCH | 68 | 7.0 | 84 | 4.0 | 0.23 | 0.78 | 58 | 5.2 | 77 | 8.3 | 0.10 | 0.32 |
| **OPEs** | | | | | | | | | | | | |
| TEP | 81 | 3.0 | 87 | 5.3 | 0.01 | 0.03 | 76 | 1.2 | 80 | 3.0 | 0.01 | 0.04 |
| TCEP | 89 | 3.1 | 97 | 2.0 | 0.02 | 0.07 | 62 | 1.5 | 66 | 3.4 | 0.03 | 0.11 |
| TPPO | 99 | 4.8 | 96 | 10 | 0.02 | 0.08 | 66 | 3.5 | 54 | 9.8 | 0.02 | 0.06 |
| TCIPP | 87 | 12 | 98 | 10 | 0.03 | 0.10 | 51 | 10 | 61 | 5.8 | 0.01 | 0.05 |
| TPP | 73 | 6.6 | 94 | 6.7 | 0.01 | 0.03 | 73 | 3.0 | 74 | 2.6 | 0.01 | 0.03 |
| TDCIPP | 81 | 7.0 | 92 | 6.1 | 0.05 | 0.18 | 50 | 5.6 | 57 | 6.1 | 0.04 | 0.14 |
| TPHP | 81 | 7.1 | 99 | 5.8 | 0.01 | 0.04 | 89 | 1.8 | 94 | 3.4 | 0.01 | 0.03 |
| TNBP | 97 | 4.0 | 93 | 8.5 | 0.01 | 0.04 | 69 | 6.3 | 66 | 2.6 | 0.01 | 0.02 |
| DCP | 90 | 5.1 | 95 | 10 | 0.03 | 0.11 | 74 | 7.8 | 60 | 1.2 | 0.02 | 0.08 |
| TBOEP | 90 | 6.9 | 97 | 2.1 | 0.02 | 0.05 | 71 | 3.3 | 61 | 4.8 | 0.01 | 0.05 |
| 2IPPDPP | 67 | 8.3 | 78 | 12 | 0.01 | 0.03 | 58 | 5.4 | 56 | 1.6 | 0.01 | 0.02 |
| RDP | 76 | 8.5 | 98 | 3.4 | 0.01 | 0.03 | 72 | 13 | 61 | 7.6 | 0.001 | 0.004 |
| 4IPPDPP | 68 | 8.2 | 97 | 2.8 | 0.01 | 0.02 | 63 | 7.9 | 63 | 2.4 | 0.01 | 0.02 |
| TCP | 94 | 6.9 | 99 | 5.9 | 0.08 | 0.26 | 80 | 9.6 | 68 | 4.6 | 0.01 | 0.03 |
| EHDPP | 84 | 9.3 | 92 | 2.5 | 0.08 | 0.27 | 75 | 6.3 | 93 | 3.0 | 0.07 | 0.22 |
| B4IPPPP | 71 | 12 | 92 | 12 | 0.02 | 0.07 | 53 | 5.4 | 62 | 4.6 | 0.01 | 0.04 |
| IDPP | 69 | 7.4 | 98 | 9.6 | 0.08 | 0.28 | 56 | 1.1 | 56 | 2.3 | 0.21 | 0.69 |
| T2IPPP | 69 | 6.8 | 95 | 12 | 0.01 | 0.04 | 90 | 2.9 | 67 | 3.9 | 0.01 | 0.05 |
| THP | 84 | 5.1 | 83 | 9.2 | 0.02 | 0.08 | 68 | 5.1 | 91 | 3.4 | 0.05 | 0.16 |
| TEHP | 90 | 5.9 | 88 | 5.1 | 0.16 | 0.52 | 96 | 8.8 | 96 | 8.4 | 0.07 | 0.23 |

*Phthalates and alternative plasticizers low level = 100 ng spiked; OPEs low level = 10 ng spiked. Phthalates and alternative plasticizers high level = 1000 ng spiked; OPEs high level = 100 ng spiked.

**Table S4.** Cleanup methodologies, instrumental techniques, and method limits of detection (mLODs) and quantification (mLOQs) of different groups of plasticizers in foodstuffs, face masks, and ambient air from other published studies.

| **Foodstuffs** | | | | | | |
| --- | --- | --- | --- | --- | --- | --- |
| **Analytes (number of compounds)** | **Matrix** | **Cleanup** | **Instrumental analysis** | **mLODs (ng/g ww)** | **mLOQs (ng/g ww)** | **Reference** |
| Phthalates (8) | Foodstuffs | Offline (GPC) | GC-MS | - | 0.20-8.00 | [1] |
| Phthalates (20) | Fatty food packaged | Offline (SPE) | LC-MS/MS | 0.02-1.60 | 0.06-5.20 | [2] |
| Phthalates (20), Alternatives (1) | Foodstuffs | Offline (Dispersive SPE) | GC-MS | 0.99-39.0 | - | [3] |
| Phthalates (6) | Foodstuffs | Online (GP-MSE) | GC-MS | 0.14-0.38 | - | [4] |
| Phthalates (7), Alternatives (1) | Meat roasted in plastic bags | Online (SPME) | GC-MS | 0.01-0.18 | - | [5] |
| Phthalates (14), Alternatives (1) | Baby foods | Offline (Dispersive SPE) | GC-MS/MS | - | 0.03-1.08 | [6] |
| Phthalates (5), Alternatives (2), Others (2) | Cereal based products | - | LC-MS/MS | 1.00-50.0 | 2.50-100 | [7] |
| Phthalates (8), Alternatives (3) | Fast food items | Offline (SPE) | GC-MS | 1.00-14.0 | - | [8] |
| Alternatives (8) | Foodstuffs | Offline (Dispersive SPE) | LC-HRMS | 0.42 (ATBC) | 0.70 (ATBC) | [9] |
| Phthalates (12), Alternatives (1) | Fish and squid | Offline (Dispersive SPE) | GC-MS | 0.50-5.00 | - | [10] |
| Phthalates (4), Alternatives (3), Others (11) | Food composites | - | LC-MS/MS | 0.10-0.30 | - | [11] |
| Phthalates (15) | Edible oils | Offline (Dispersive SPE) | GC-MS/MS | 0.01-5.17 | 0.04-17.2 | [12] |
| Phthalates (22) | Foodstuffs | - | GC-MS | - | - | [13] |
| Phthalates (6) | Foodstuffs | Offline (SPE) | GC-MS | 1.30-3.30 | 4.30-11.0 | [14] |
| OPEs (9) and their metabolites (11) | Foodstuffs | Offline (Dispersive SPE) | LC-MS/MS | 0.004-3.30 | - | [15] |
| OPEs (14) | Foodstuffs | Offline (Dispersive SPE and SPE) | GC-MS/MS | - | 0.002-62.0 | [16] |
| OPEs (15) | Foodstuffs | Offline (Dispersive SPE and SPE) | LC-MS/MS | 0.02-0.17 | - | [17] |
| OPEs (10) | Foodstuffs | Offline (Dispersive SPE) | LC-MS/MS | - | 0.07-0.42 | [18] |
| OPEs (11) | Foodstuffs | Offline (Dispersive SPE) | LC-MS/MS | 0.003-0.107 | 0.01-0.36 | [19] |
| OPEs (8) | Foodstuffs | Offline (Dispersive SPE and SPE) | GC-MS/MS | - | 0.07-0.69 | [20] |
| OPEs (19 targeted and 2 suspect) | Foodstuffs | Offline (Dispersive SPE) | LC-HRMS | 0.001-0.058 | 0.002-0.194 | [21] |
| OPEs (25) | Foodstuffs | Offline (SPE) | LC-MS/MS |  | 0.004-1.32 | [22] |
| OPEs (19), antioxidants (4) | Takeaway food | Offline (Dispersive SPE) | LC-MS/MS | - | 0.003-1.684 | [23] |
| Phthalates (10), Alternatives (4) | Foodstuffs | Online (TFC) | LC-MS/MS | 0.01-2.08 | 0.04-6.92 | Our study |
| OPEs (20) |  |  |  | 0.001-0.21 | 0.004-0.69 |  |
| **Plastic materials** | | | | | | |
| **Analytes (number of compounds)** | **Matrix** | **Cleanup** | **Instrumental analysis** | **mLODs (ng/g)** | **mLOQs (ng/g)** | **Reference** |
| Phthalates (8) | Face masks | - | GC-HRMS | 0.016-10.0 ng/sample | - | [24] |
| Phthalates (5) | Face masks | - | GC-MS | 1.10-3.54 | 3.66-11.8 | [25] |
| Phthalates (12) | Face masks | - | GC-MS | 5.10-26.5 | - | [26] |
| Phthalates (7) | Face masks | - | GC-MS | 0.073-3.41 | 0.25-10.8 | [27] |
| Phthalates (11) | Face masks | - | GC-MS | 10.0-30.0 | 30.0-80.0 | [28] |
| Phthalates (6) | Face masks | - | LC-HRMS | - | 0.02-0.05 | [29] |
| OPEs (11) |  |  |  | - | 0.01-0.07 |  |
| Phthalates (10), Alternatives (4) | Face masks | Online (TFC) | LC-MS/MS | 0.003-0.23 | 0.01-0.77 | Our study |
| OPEs (16) |  |  |  | 0.002-0.30 | 0.01-0.99 |  |
| **Air samples** | | | | | | |
| **Analytes (number of compounds)** | **Matrix** | **Cleanup** | **Instrumental analysis** | **mLODs (ng/m^3^)** | **mLOQs (ng/m^3^)** | **Reference** |
| Phthalates (5), Alternatives (1), Others (13) | QFFs (Outdoor air) | - | GC-MS | 0.002-0.014 | 0.004-0.068 | [30] |
| OPEs (7) |  |  |  | 0.001-0.004 | 0.003-0.009 |  |
| Phthalates (12), Others (26) | QFFs (Outdoor air) | Offline (Dispersive SPE) | GC-MS/MS | 0.003-1.13 | 0.005-1.79 | [31] |
| OPEs (12) |  |  |  | 0.003-0.16 | 0.005-0.30 |  |
| Phthalates (6), Alternatives (1), Others (7) | QFFs (Outdoor air) | - | LC-MS/MS | 0.03-1.15 | 0.09-3.85 | [32] |
| Phthalates (8), Others (12) | GFFs (Indoor air) | Offline (SPE) | GC-MS | 0.007-0.268 | - | [33] |
| OPEs (9) |  |  |  | 0.003-0.012 | - |  |
| Phthalates (14) | GFFs (Outdoor air) | - | GC-MS | 0.007-0.13 | - | [34] |
| Phthalates (7), Alternatives (8) | QFFs (Indoor air) | - | GC-MS | 0.014-13.0 | - | [35] |
| Phthalates (8), Alternatives (3) | QFFs (Indoor air) | Online (TFC) | LC-MS/MS | 0.001-0.93 | 0.004-3.10 | Our study |
| OPEs (18) |  |  |  | 0.002-0.08 | 0.01-0.27 |  |

**Table S5.** Internal standard (IS) recovery ranges (%) in the different matrices analyzed.

| **IS** | **Ranges (%)** |
| --- | --- |
| d_4_-DMP | 66-97 |
| d_4_-DEP | 79-90 |
| d_4_-DBP | 64-87 |
| d_4_-BBzP | 64-85 |
| d_4_-DCHP | 80-83 |
| d_4_-DHexP | 82-94 |
| d_4_-DEHP | 67-92 |
| d_4_-DnOP | 87-93 |
| d_3_-ATBC | 91-97 |
| d_15_-TEP | 60-64 |
| d_12_-TCEP | 64-69 |
| d_21_-TPP | 66-71 |
| d_15_-TDCIPP | 65-74 |
| d_15_-TPHP | 62-64 |
| d_27_-TNBP | 67-78 |
| ^13^C_2_-TBOEP | 62-84 |
| d_51_-TEHP | 61-84 |

**Table S6.** Recoveries, relative standard deviations (RSDs), and sensitivity parameters of selected plasticizers in face masks and QFFs.

| **Analyte** | **Face masks** | | | | | | **QFFs** | | | | | |  |
| --- | --- | --- | --- | --- | --- | --- | --- | --- | --- | --- | --- | --- | --- |
|  | **Low spike - 150 ng (n = 4)** | | **High spike - 1500 ng (n = 4)** | | **mLOD (ng g^-1^)** | **mLOQ (ng g^-1^)** | **Low spike - 50 ng (n = 3)** | | **High spike - 500 ng (n = 3)** | | **mLOD (ng m^-3^)** | **mLOQ (ng m^-3^)** |  |
|  |  |  |  |  |  |  |  |  |  |  |  |  |  |
|  | **Recovery (%)** | **RSD (%)** | **Recovery (%)** | **RSD (%)** |  |  | **Recovery (%)** | **RSD (%)** | **Recovery (%)** | **RSD (%)** |  |  |  |
| **PHTHALATES** | | | | | | | | | | | | |  |
| DMP | 41 | 12 | 51 | 2.9 | 0.02 | 0.06 | - | - | - | - | - | - |  |
| DEP | - | - | - | - | - | - | 125 | 13 | 107 | 1.5 | 0.03 | 0.10 |  |
| DiBP | 89 | 1.5 | 120 | 3.5 | 0.005 | 0.02 | 49** | 3.2** | 55** | 4.8** | 0.01** | 0.03** |  |
| BBzP | 55 | 6.3 | 82 | 4.4 | 0.01 | 0.02 | 56 | 8.0 | 59 | 5.3 | 0.01 | 0.03 |  |
| DnBP | 88 | 17 | 74 | 4.0 | 0.003 | 0.01 | - | - | - | - | - | - |  |
| DCHP | 73 | 3.2 | 73 | 8.8 | 0.003 | 0.01 | - | - | - | - | - | - |  |
| DHexP | 72 | 4.5 | 79 | 8.5 | 0.01 | 0.03 | 65 | 6.7 | 55 | 4.2 | 0.01 | 0.02 |  |
| DEHP | 67 | 11 | 54 | 14 | 0.05 | 0.17 | 40 | 19 | 58 | 10 | 0.02 | 0.06 |  |
| DnOP | 62 | 5.6 | 64 | 12 | 0.14 | 0.45 | 64 | 6.5 | 60 | 9.2 | 0.07 | 0.22 |  |
| DiNP | 58 | 13 | 68 | 13 | 0.23 | 0.77 | 53 | 3.5 | 76 | 4.6 | 0.93 | 3.10 |  |
| DiDP | 110 | 19 | 115 | 6.0 | 0.14 | 0.48 | - | - | - | - | - | - |  |
| **ALTERNATIVE PLASTICIZERS** | | | | | | | | | | | | |  |
| ATBC | 95 | 13 | 84 | 6.9 | 0.003 | 0.01 | 63 | 2.2 | 58 | 6.1 | 0.003 | 0.01 |  |
| DEHA | - | 7.4 | 63 | 15 | 0.02 | 0.08 | 57 | 8.9 | 60 | 11 | 0.10 | 0.35 |  |
| DINA | 73 | 10 | 98 | 16 | 0.06 | 0.19 | 80 | 2.1 | 82 | 8.7 | 0.10 | 0.33 |  |
| DINCH | 72 | 19 | 100 | 20 | 0.09 | 0.29 | - | - | - | - | - | - |  |
| **OPEs*** | | | | | | | | | | | | |  |
| TEP | 54 | 7.5 | 40 | 10 | 0.01 | 0.03 | - | - | - | - | - | - |  |
| TCEP | 56 | 8.4 | 70 | 5.0 | 0.02 | 0.06 | 63 | 3.4 | 61 | 0.1 | 0.01 | 0.04 |  |
| TPPO | 85 | 10 | 91 | 13 | 0.11 | 0.38 | 61 | 8.7 | 66 | 17 | 0.004 | 0.01 |  |
| TCIPP | 89 | 16 | 96 | 6.2 | 0.03 | 0.09 | 64 | 3.9 | 57 | 9.6 | 0.02 | 0.08 |  |
| TPP | 85 | 3.2 | 65 | 4.5 | 0.01 | 0.04 | - | - | - | - | - | - |  |
| TDCIPP | 105 | 1.2 | 92 | 3.4 | 0.08 | 0.27 | 83 | 3.1 | 79 | 1.6 | 0.03 | 0.11 |  |
| TPHP | 52 | 6.3 | 94 | 6.3 | 0.01 | 0.03 | 83 | 2.4 | 77 | 2.8 | 0.004 | 0.01 |  |
| TNBP | 59 | 10 | 75 | 8.7 | 0.003 | 0.01 | 85 | 9.3 | 69 | 12 | 0.002 | 0.01 |  |
| DCP | 88 | 2.0 | 85 | 7.3 | 0.01 | 0.03 | 117 | 0.8 | 98 | 0.8 | 0.01 | 0.02 |  |
| TBOEP | - | - | - | - | - | - | 64 | 2.4 | 62 | 3.4 | 0.004 | 0.01 |  |
| 2IPPDPP | 97 | 4.1 | 105 | 7.7 | 0.002 | 0.01 | 82 | 2.1 | 88 | 9.9 | 0.002 | 0.01 |  |
| RDP | - | - | - | - | - | - | 91 | 3.2 | 96 | 6.9 | 0.01 | 0.03 |  |
| 4IPPDPP | - | - | - | - | - | - | 88 | 3.6 | 101 | 12 | 0.002 | 0.01 |  |
| TCP | 66 | 1.1 | 66 | 1.1 | 0.003 | 0.01 | 98 | 2.3 | 89 | 6.1 | 0.002 | 0.01 |  |
| EHDPP | - | - | - | - | - | - | 83 | 2.8 | 85 | 11 | 0.02 | 0.07 |  |
| B4IPPPP | 94 | 5.2 | 110 | 3.9 | 0.004 | 0.01 | 63 | 4.9 | 74 | 9.9 | 0.003 | 0.01 |  |
| IDPP | 78 | 3.6 | 78 | 14 | 0.30 | 0.99 | - | - | - | - | - | - |  |
| T2IPPP | - | - | - | - | - | - | 117 | 6.7 | 117 | 6.7 | 0.01 | 0.05 |  |
| THP | 74 | 7.2 | 56 | 20 | 0.09 | 0.30 | 104 | 5.0 | 97 | 5.5 | 0.02 | 0.08 |  |
| TEHP | 41 | 2.3 | 97 | 15 | 0.05 | 0.15 | 86 | 9.0 | 75 | 1.5 | 0.08 | 0.27 |  |

Compounds with unacceptable analytical parameters were excluded from the methodology and denoted with a dash.

*Face masks: OPEs low level = 25 ng spiked; OPEs high level = 250 ng spiked. QFFs: OPEs low level = 20 ng spiked; OPEs high level = 100 ng spiked. **DiBP+DnBP.

**Table S7.** Plasticizer results from the developed method applied to selected real samples.

| **Analyte** | **Low fat foodstuffs (ng/g ww)** | | | **High fat foodstuffs (ng/g ww)** | | | **Face masks (ng/g)** | | | **QFFs (ng/m^3^)** | | |
| --- | --- | --- | --- | --- | --- | --- | --- | --- | --- | --- | --- | --- |
|  | **Breakfast cereals** | **Rice** | **Sweetener** | **Beef** | **Salmon** | **Yogurt** | **Cloth reusable mask** | **FFP2 mask** | **FFP3 mask** | **Motorcycle repair garage** | **Art studio** | **Pottery studio** |
| **PHTHALATES** | | | | | | | | | | | | |
| DMP | nd | nd | nd | 27 | 5.55 | 1.75 | 11.0 | nq | 6.43 | - | - | - |
| DEP | 19.3 | nd | 71.2 | 114 | 3.51 | nd | - | - | - | 151 | 22.1 | 69.8 |
| DiBP | nd | nd | nd | nd | nd | nd | 54.5 | 819 | 1028 | 55.7* | 301* | 385* |
| BBzP | nd | nd | 9.00 | nd | nd | nd | 24.9 | 1.73 | 125 | nq | nq | nq |
| DnBP | - | - | - | - | - | - | 72.0 | 182 | nq | - | - | - |
| DCHP | nd | nd | nd | nd | nd | nd | nq | nd | nq | 0.03 | 0.02 | 0.01 |
| DHexP | nd | nd | nd | nd | nd | nd | nd | nd | 4.27 | nq | 0.39 | 0.17 |
| DEHP | 71.4 | nd | nd | nd | nd | nd | 1391 | 1766 | 232 | 45.5 | 27.6 | 27.7 |
| DnOP | nd | nd | nd | nd | nd | nd | nd | nd | nd | nd | nd | nd |
| DiNP | 459 | 472 | 205 | nd | nd | 147 | 703 | 2094 | 30.1 | 6.39 | 9.69 | 18.0 |
| DiDP | nd | nd | nd | nd | nd | nd | 384 | 707 | 2933 | - | - | - |
| **TOTAL** | **549** | **472** | **285** | **141** | **9.05** | **149** | **2641** | **5570** | **4358** | **259** | **360** | **500** |
| **ALTERNATIVE PLASTICIZERS** | | | | | | | | | | | | |
| ATBC | 555 | 166 | 21563 | nd | 3.75 | nd | 122 | 304 | 0.33 | 7.91 | 5.19 | 6.48 |
| DEHA | 48.9 | nd | nd | 81.9 | nd | 20.7 | 39.7 | 100 | 23.8 | 6.38 | 5.57 | 6.55 |
| DINA | nd | nd | nd | nd | nd | nd | nd | 391 | 5.40 | nq | nq | nq |
| DINCH | nd | 261 | nd | nd | nd | nd | 72.6 | nd | 8.51 | - | - | - |
| **TOTAL** | **604** | **427** | **21563** | **81.9** | **3.75** | **20.7** | **235** | **795** | **38.0** | **14.3** | **10.8** | **13.0** |
| **OPEs** | | | | | | | | | | | | |
| TEP | nd | nd | nd | nd | nd | nd | 136 | 325 | 0.27 | - | - | - |
| TCEP | nd | nd | nd | nd | nd | nd | nd | nd | 3.67 | 1.64 | 17.8 | 25.5 |
| TPPO | nd | nd | nd | nd | 20.0 | nd | nd | nd | nq | 0.18 | 0.04 | 0.03 |
| TCIPP | nd | nd | nd | nd | 2.59 | nd | nd | 5.13 | nq | 29.7 | 30.7 | 28.1 |
| TPP | nd | nd | nd | nd | nd | nd | nd | nd | nd | - | - | - |
| TDCIPP | nd | nd | nd | nd | nd | nd | 2.19 | 2.04 | nq | 0.51 | 0.28 | 1.61 |
| TPHP | nd | 3.45 | nd | 0.25 | 0.70 | 1.08 | 33.8 | 13.4 | 0.13 | 0.27 | 0.22 | 0.28 |
| TNBP | nd | 2.20 | 117 | nd | nd | nd | 8.57 | 53.0 | 10.9 | 3.41 | 3.02 | 1.44 |
| DCP | 1.10 | 18.7 | nd | nd | 0.81 | 0.17 | 4.80 | nd | nq | 0.09 | 0.07 | 0.08 |
| TBOEP | nd | nd | nd | nd | nd | nd | - | - | - | 0.30 | 0.05 | 0.09 |
| 2IPPDPP | nd | nd | nd | nd | nd | 0.34 | nd | nd | nq | 0.16 | 0.09 | 0.16 |
| RDP | nd | nd | nd | nd | nd | nd | - | - | - | nd | nd | nd |
| 4IPPDPP | nd | nd | nd | nd | nd | nd | - | - | - | 0.06 | 0.05 | 0.02 |
| TCP | nd | 0.48 | nd | nd | nd | nd | nd | nd | nd | 0.31 | 0.14 | 0.03 |
| EHDPP | 19.9 | 38.1 | nd | 0.89 | nd | nd | - | - | - | 0.17 | 0.61 | 1.05 |
| B4IPPPP | nd | nd | nd | nd | nd | nd | nd | nd | nq | 0.09 | 0.04 | 0.17 |
| IDPP | nd | nd | nd | nd | nd | nd | nd | nd | nd | nd | nd | nd |
| T2IPPP | nd | nd | nd | nd | nd | nd | nd | nd | nd | nd | nd | nd |
| THP | nd | nd | nd | nd | nd | nd | nd | nd | nd | nq | nd | nd |
| TEHP | nd | nd | nd | nd | nd | nd | 66.5 | 14.7 | 2938 | nd | nd | nd |
| **TOTAL** | **21.0** | **63.0** | **117** | **1.14** | **24.1** | **1.60** | **252** | **413** | **2953** | **36.9** | **53.1** | **58.6** |
| **TOTAL ADDIT.** | **1174** | **962** | **21965** | **224** | **36.9** | **171** | **3128** | **6778** | **7349** | **310** | **424** | **572** |

*DiBP+DnBP.

nd: not detected, below mLOD; nq: not quantifiable, below mLOQ.


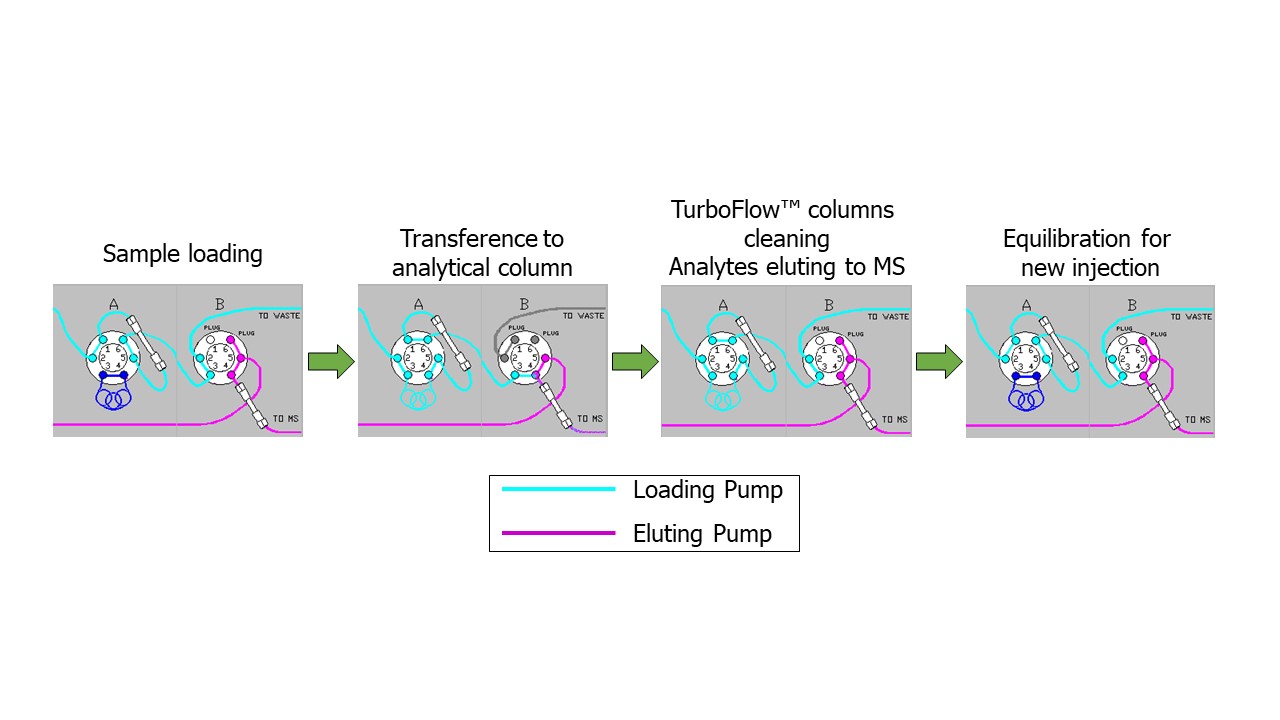


**Figure S1.** TurboFlow™ workflow description of the Focus mode.

**References**

1. Van Holderbeke M, Geerts L, Vanermen G, Servaes K, Sioen I, De Henauw S, Fierens T (2014) Determination of contamination pathways of phthalates in food products sold on the Belgian market. Environ Res 134:345–352. https://doi.org/10.1016/j.envres.2014.08.012

2. Fan J, Wu L, Wang X, Huang X, Jin Q, Wang S (2012) Determination of the migration of 20 phthalate esters in fatty food packaged with different materials by solid-phase extraction and UHPLC–MS/MS. Analytical Methods 4:4168. https://doi.org/10.1039/c2ay25916h

3. Cao X-L, Zhao W, Dabeka R (2015) Di-(2-ethylhexyl) adipate and 20 phthalates in composite food samples from the 2013 Canadian Total Diet Study. Food Additives & Contaminants: Part A 32:1893–1901. https://doi.org/10.1080/19440049.2015.1079742

4. He M, Yang C, Geng R, Zhao X, Hong L, Piao X, Chen T, Quinto M, Li D (2015) Monitoring of phthalates in foodstuffs using gas purge microsyringe extraction coupled with GC–MS. Anal Chim Acta 879:63–68. https://doi.org/10.1016/j.aca.2015.02.066

5. Moreira MA, André LC, Cardeal Z de L (2015) Analysis of plasticiser migration to meat roasted in plastic bags by SPME–GC/MS. Food Chem 178:195–200. https://doi.org/10.1016/j.foodchem.2015.01.078

6. Socas-Rodríguez B, González-Sálamo J, Herrera-Herrera A V., Santana-Mayor Á, Hernández-Borges J (2018) Determination of phthalic acid esters in different baby food samples by gas chromatography tandem mass spectrometry. Anal Bioanal Chem 410:5617–5628. https://doi.org/10.1007/s00216-018-0977-y

7. García Ibarra V, Sendón R, Bustos J, Paseiro Losada P, Rodríguez Bernaldo de Quirós A (2019) Estimates of dietary exposure of Spanish population to packaging contaminants from cereal based foods contained in plastic materials. Food and Chemical Toxicology 128:180–192. https://doi.org/10.1016/j.fct.2019.04.003

8. Edwards L, McCray NL, VanNoy BN, Yau A, Geller RJ, Adamkiewicz G, Zota AR (2022) Phthalate and novel plasticizer concentrations in food items from U.S. fast food chains: a preliminary analysis. J Expo Sci Environ Epidemiol 32:366–373. https://doi.org/10.1038/s41370-021-00392-8

9. Zhang Y, Li J, Su G (2022) Comprehensively screening of citric acid ester (CAE) plasticizers in Chinese foodstuffs, and the food-based assessment of human exposure risk of CAEs. Science of The Total Environment 817:152933. https://doi.org/10.1016/j.scitotenv.2022.152933

10. Sambolino A, Ortega-Zamora C, González-Sálamo J, Dinis A, Cordeiro N, Canning-Clode J, Hernández-Borges J (2022) Determination of phthalic acid esters and di(2-ethylhexyl) adipate in fish and squid using the ammonium formate version of the QuEChERS method combined with gas chromatography mass spectrometry. Food Chem 380:132174. https://doi.org/10.1016/j.foodchem.2022.132174

11. Tian L, Zheng J, Pineda M, Yargeau V, Furlong D, Chevrier J, Bornman R, Obida M, Gates Goodyer C, Bayen S (2022) Targeted screening of 11 bisphenols and 7 plasticizers in food composites from Canada and South Africa. Food Chem 385:132675. https://doi.org/10.1016/j.foodchem.2022.132675

12. Wang X, Sun X, Wang X, Qi X, Wang D, Jiang J, Mao J, Ma F, Yu L, Zhang L, Li P (2022) Determination of 15 phthalic acid esters based on GC–MS/MS coupled with modified QuEChERS in edible oils. Food Chem X 16:100520. https://doi.org/10.1016/j.fochx.2022.100520

13. Ma G, Ma B, Wang L, Tao W (2022) Occurrence and dietary exposure risks of phthalate esters in food in the typical valley city Xi’an, Northwest China. Environmental Science and Pollution Research 29:31426–31440. https://doi.org/10.1007/s11356-022-18592-6

14. Fu L, Song S, Luo X, Luo Y, Guo C, Liu Y, Luo X, Zeng L, Tan L (2023) Unraveling the contribution of dietary intake to human phthalate internal exposure. Environmental Pollution 337:122580. https://doi.org/10.1016/j.envpol.2023.122580

15. He C, Wang X, Tang S, Thai P, Li Z, Baduel C, Mueller JF (2018) Concentrations of Organophosphate Esters and Their Specific Metabolites in Food in Southeast Queensland, Australia: Is Dietary Exposure an Important Pathway of Organophosphate Esters and Their Metabolites? Environ Sci Technol 52:12765–12773. https://doi.org/10.1021/acs.est.8b03043

16. Poma G, Sales C, Bruyland B, Christia C, Goscinny S, Van Loco J, Covaci A (2018) Occurrence of Organophosphorus Flame Retardants and Plasticizers (PFRs) in Belgian Foodstuffs and Estimation of the Dietary Exposure of the Adult Population. Environ Sci Technol 52:2331–2338. https://doi.org/10.1021/acs.est.7b06395

17. Wang Y, Kannan K (2018) Concentrations and Dietary Exposure to Organophosphate Esters in Foodstuffs from Albany, New York, United States. J Agric Food Chem 66:13525–13532. https://doi.org/10.1021/acs.jafc.8b06114

18. Ding J, Deng T, Xu M, Wang S, Yang F (2018) Residuals of organophosphate esters in foodstuffs and implication for human exposure. Environmental Pollution 233:986–991. https://doi.org/10.1016/j.envpol.2017.09.092

19. Zhao L, Jian K, Su H, Zhang Y, Li J, Letcher RJ, Su G (2019) Organophosphate esters (OPEs) in Chinese foodstuffs: Dietary intake estimation via a market basket method, and suspect screening using high-resolution mass spectrometry. Environ Int 128:343–352. https://doi.org/10.1016/j.envint.2019.04.055

20. Gbadamosi MR, Abdallah MA-E, Harrad S (2022) Organophosphate esters in UK diet; exposure and risk assessment. Science of The Total Environment 849:158368. https://doi.org/10.1016/j.scitotenv.2022.158368

21. Bi R, Su G (2023) Dietary intake assessment of known and unknown organophosphate esters (OPEs) in foodstuffs via high-resolution mass spectrometry. Science of The Total Environment 854:158452. https://doi.org/10.1016/j.scitotenv.2022.158452

22. Chen X, Liang X, Yang J, Yuan Y, Xiao Q, Su Z, Chen Y, Lu S, Wang L (2023) High-resolution mass spectrometry-based screening and dietary intake assessment of organophosphate esters in foodstuffs from South China. Science of The Total Environment 905:167169. https://doi.org/10.1016/j.scitotenv.2023.167169

23. Zhou R, Geng J, Jiang J, Shao B, Wang B, Wang Y, Li M (2024) Emerging organophosphite and organophosphate esters in takeaway food and the implications for human exposure. Environmental Science and Pollution Research 31:32588–32598. https://doi.org/10.1007/s11356-024-33413-8

24. Wang X, Okoffo ED, Banks AP, Li Y, Thomas K V., Rauert C, Aylward LL, Mueller JF (2022) Phthalate esters in face masks and associated inhalation exposure risk. J Hazard Mater 423:127001. https://doi.org/10.1016/j.jhazmat.2021.127001

25. Shende N, Hippargi G, Gurjar S, Kumar AR, Rayalu S (2022) Occurrence of phthalates in facemasks used in India and its implications for human exposure. Int J Environ Health Res 1–17. https://doi.org/10.1080/09603123.2022.2135691

26. Xie H, Han W, Xie Q, Xu T, Zhu M, Chen J (2022) Face mask—A potential source of phthalate exposure for human. J Hazard Mater 422:126848. https://doi.org/10.1016/j.jhazmat.2021.126848

27. Cao J, Shi Y, Yan M, Zhu H, Chen S, Xu K, Wang L, Sun H (2023) Face Mask: As a Source or Protector of Human Exposure to Microplastics and Phthalate Plasticizers? Toxics 11:87. https://doi.org/10.3390/toxics11020087

28. Leoni C, Majorani C, Cresti R, Marcello I, Berardi E, Fava L, Attias L, D’Ilio S (2023) Determination and risk assessment of phthalates in face masks. An Italian study. J Hazard Mater 443:130176. https://doi.org/10.1016/j.jhazmat.2022.130176

29. Wang C, Su Z-H, He M-J (2023) Dynamic variation and inhalation exposure of organophosphates esters and phthalic acid esters in face masks. Environmental Pollution 316:120703. https://doi.org/10.1016/j.envpol.2022.120703

30. Maceira A, Pecikoza I, Marcé RM, Borrull F (2020) Multi-residue analysis of several high-production-volume chemicals present in the particulate matter from outdoor air. A preliminary human exposure estimation. Chemosphere 252:126514. https://doi.org/10.1016/j.chemosphere.2020.126514

31. Sánchez-Piñero J, Moreda-Piñeiro J, Moscoso-Pérez C, FernándezGonzález V, Prada-Rodríguez D, López-Mahía P (2021) Development and validation of a multi-pollutant method for the analysis of polycyclic aromatic hydrocarbons, synthetic musk compounds and plasticizers in atmospheric particulate matter (PM2.5). Talanta Open 4:100057. https://doi.org/10.1016/j.talo.2021.100057

32. Patnana DP, Chandra BP, Chaudhary P, Sinha B, Sinha V (2022) Optimized LC-MS/MS method for simultaneous determination of endocrine disruptors and PAHs bound to PM2.5: Sources and health risk in Indo-Gangetic Plain. Atmos Environ 290:119363. https://doi.org/10.1016/j.atmosenv.2022.119363

33. Wang Y, Zhang Z, Xu Y, Rodgers TFM, Ablimit M, Li J, Tan F (2023) Identifying the contributions of root and foliage gaseous/particle uptakes to indoor plants for phthalates, OPFRs and PAHs. Science of The Total Environment 883:163644. https://doi.org/10.1016/j.scitotenv.2023.163644

34. Lu H, Chen D, Zhu Z, Yang L, Huang L, Xu C, Lu Y (2023) Atmospheric phthalate esters in a multi-function area of Hangzhou: Temporal variation, gas/particle phase distribution, and population exposure risk. Science of The Total Environment 894:163987. https://doi.org/10.1016/j.scitotenv.2023.163987

35. Huo C-Y, Li W-L, Liu L-Y, Sun Y, Guo J-Q, Wang L, Hung H, Li Y-F (2023) Seasonal variations of airborne phthalates and novel non-phthalate plasticizers in a test residence in cold regions: Effects of temperature, humidity, total suspended particulate matter, and sources. Science of The Total Environment 863:160852. https://doi.org/10.1016/j.scitotenv.2022.160852
